# Expanding the Marine Virosphere Using Metagenomics

**DOI:** 10.1371/journal.pgen.1003987

**Published:** 2013-12-12

**Authors:** Carolina Megumi Mizuno, Francisco Rodriguez-Valera, Nikole E. Kimes, Rohit Ghai

**Affiliations:** Evolutionary Genomics Group, Departamento de Producción Vegetal y Microbiología, Universidad Miguel Hernández, San Juan de Alicante, Alicante, Spain; Institut Pasteur, France

## Abstract

Viruses infecting prokaryotic cells (phages) are the most abundant entities of the biosphere and contain a largely uncharted wealth of genomic diversity. They play a critical role in the biology of their hosts and in ecosystem functioning at large. The classical approaches studying phages require isolation from a pure culture of the host. Direct sequencing approaches have been hampered by the small amounts of phage DNA present in most natural habitats and the difficulty in applying meta-omic approaches, such as annotation of small reads and assembly. Serendipitously, it has been discovered that cellular metagenomes of highly productive ocean waters (the deep chlorophyll maximum) contain significant amounts of viral DNA derived from cells undergoing the lytic cycle. We have taken advantage of this phenomenon to retrieve metagenomic fosmids containing viral DNA from a Mediterranean deep chlorophyll maximum sample. This method allowed description of complete genomes of 208 new marine phages. The diversity of these genomes was remarkable, contributing 21 genomic groups of tailed bacteriophages of which 10 are completely new. Sequence based methods have allowed host assignment to many of them. These predicted hosts represent a wide variety of important marine prokaryotic microbes like members of SAR11 and SAR116 clades, *Cyanobacteria* and also the newly described low GC *Actinobacteria*. A metavirome constructed from the same habitat showed that many of the new phage genomes were abundantly represented. Furthermore, other available metaviromes also indicated that some of the new phages are globally distributed in low to medium latitude ocean waters. The availability of many genomes from the same sample allows a direct approach to viral population genomics confirming the remarkable mosaicism of phage genomes.

## Author Summary

Prokaryotic species contain extremely large gene pools (pan-genome) the study of which has been constrained by the difficulties in getting enough cultivated representatives of most of them. The situation of their viruses, also known as phages, that provide part of this genomic diversity and preserve it, is even worse. Here we have found a way to bypass the limitation imposed by pure culture to retrieve phage genomes. We obtained large insert clones (fosmids) from natural communities that are undergoing active viral attack. This has allowed us to triple the number of genomes of marine phages and could be similarly applied to other habitats, shedding light into the biology of the most numerous and least known biological entities on the planet. They exhibit a remarkable degree of variation at one single geographic site but some seem also to be prevalent worldwide. Their frequent mosaicism indicates a high level of promiscuity that goes beyond the already remarkable hybrid nature of prokaryotic genomes.

## Introduction

Prokaryotic viruses, often referred to as phages, are one of the largest reservoirs of underexplored genetic diversity on Earth. They are more numerous than any other biological form on the planet, and the astronomical values put forward for their numbers are to the tune of 10^30^, difficult to comprehend even by metaphoric abstractions [1]. Such estimates have contributed to an increasing appreciation of the role of this poorly charted component in the global carbon and energy cycling in the oceans [2], [3]. The high prevalence of phages in the environment also raises important questions about their local and global population diversity, the dynamics of interaction within themselves and their hosts, and the evolutionary implications of these relationships [4], [5].

A critical bottleneck for the study of phages is the need to obtain their hosts in axenic cultures before they themselves can be cultured. Consequently, as most marine bacteria remain uncultured [6]–[8], so too do their phages. Obtaining genomic DNA for uncultured microbes has been relatively easy, and sequencing of numerous oceanic metagenomes and single cell genomes have provided an extraordinarily detailed view of the real world of marine microbes [9]–[14]. Similar progress, though, has been elusive for marine phages. Even though they are estimated to be 10-fold as numerous as prokaryotic cells, recovering viral DNA in amounts sufficient for sequencing has proven difficult although recently methods have been devised to improve the process [15]–[17]. Phage genomes are much smaller than cellular ones and total phage DNA per volume is relatively low [18] compared to their cellular hosts. As a result, DNA amplification is normally a necessary step before metavirome sequencing, which probably biases the product significantly [19], [20]. Still, with all these caveats, the nascent field of marine metaviromics has provided an insight into the marine viral world [21]–[26]. Therefore, most of our knowledge about complete marine phages' genomes stems from cultured representatives [27]–[30], isolated only because of success in culturing the host, which themselves, in several cases took painstaking years to be adapted for growth in the laboratory e.g. *Ca.* Pelagibacter [31], [32].

Cloning of environmental DNA into fosmids, used successfully for studying prokaryotic genomic fragments of uncultured microbes [9], [10], has opened an alternative to obtain complete genomes of phages [18], side-stepping completely the previously mandatory availability of a cultivated host. This has been possible due to the observation that inserts cloned in fosmids from metagenomic DNA have a significant representation of phage genomic fragments [9], [10]. Actually, a replicating phage in the course of its natural lytic cycle in a cell, provides a natural amplification that is reminiscent of laboratory cloning or other methods of genome amplification, such as multiple displacement amplification (MDA) [33]. Formerly, metagenomic fosmids have been shown to capture major marine phage lineages like cyanophages [10], [18] and the SAR11 viruses [32].

The deep chlorophyll maximum (DCM) is the site of maximal phototrophic cell density in oligotrophic open ocean waters. It is a seasonal phenomenon in temperate waters forming at the middle of the photic zone during the summer stratification of the water column [34], [35] and a permanent feature in tropical latitudes. Supported by the high number of microbial cells, the number of infecting phages is also expected to be high. We have sequenced and assembled ∼6000 metagenomic fosmids obtained from the Mediterranean DCM (MedDCM) cellular fraction (>0.2 µm). Among them more than a thousand genomic contigs were derived from marine phages that were actively replicating and are described here. Two hundred and eight of them represented novel complete genomes, and some were very different from any phage known to date. Furthermore, the examination of the genomes has allowed assigning putative hosts to many of these previously unknown phages. This collection also provides a unique opportunity to examine concurrent phages from the same natural habitat, *en masse*. The sequences reveal the existence of multiple, highly related coexisting lineages for each phage type, likely matching or exceeding the multiple prokaryotic lineages of their host genomes [36]. From the same site a metavirome (from the viral size fraction) has also been directly sequenced by Illumina (MedDCM-Vir) to assess the relevance of these phages in the viral sized fraction.

## Results

### General Features of Viral Contigs

From the sequenced fosmids, we manually selected 1148 virus-like contigs (size range 5–48 Kb, average size 23 Kb, GC% range 27–57) based on their resemblance to known phages and/or presence of key phage genes using the Phage Orthologous Groups [37] (see Materials and Methods). As is typical for viral genomes, nearly half of the proteins from this collection of fosmid contigs (40%) did not present any hits to the NR database, reflecting the novelty of the phage genomes described here. Thirty six percent were similar only to hypothetical proteins. Of the remaining 24% that could be attributed a function, most (19%) were clearly phage-related, 1% were cellular-like (host) proteins and 4% were unclassified. Among host-related genes, we identified auxiliary metabolic genes (AMGs) commonly found in phages such as photosystem related genes (*psbA*, *psbD*), 6-phosphogluconate dehydrogenase (*gnd*), Glucose-6-phosphate 1-dehydrogenase (*zwf*) and transaldolase (talC) [30], [38], [39].

**Figure 1:**
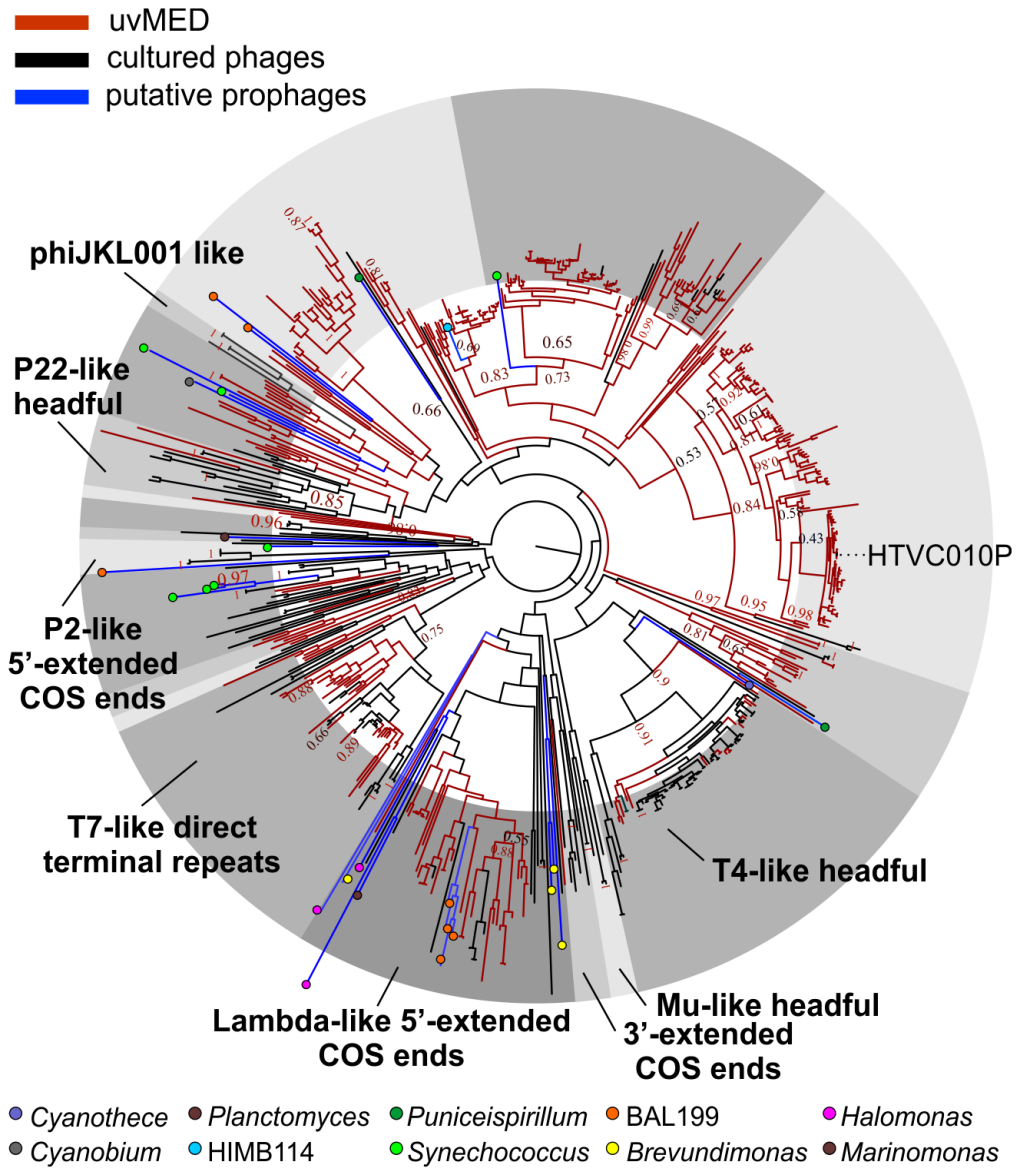
Terminase phylogeny. Maximum-likelihood tree of known representative terminase genes along with all identified terminases in the uncultured MedDCM viral contigs (uvMED) and putative prophage terminases (401 uvMED, 631 in total). Branches in the tree are colored according to the following codes: *black*, sequences from cultured phages; *blue*, putative prophage derived sequences; *red*, uncultured phage terminases obtained in this study. Known terminase types are labeled in bold. The microbes from which the putative prophage terminases were identified are marked by colored circles in the tree and a key is provided below. Bootstrap support of nodes is indicated on selected nodes. The terminase of pelagiphage HTVC010P is also labeled.

The presence of a large number of predicted proteins characteristic of tailed phages (e.g. terminase, tape measure protein, tail formation and baseplate related proteins) indicated that 935 contigs clearly originated from the order Caudovirales. For the remaining contigs, in which these specific genes could not be identified, further comparisons suggested that they were also tailed bacteriophages. Given that these contigs could be reliably assigned to head-tail phages and are derived from the cellular fraction (between 5 and 0.2 µm) selective for prokaryotic cells, we have focused this work only on tailed phages and not on other types of viruses (e.g. eukaryotic viruses) that might also be present in the fosmid library. Phylogeny of the essential terminase gene has been used to resolve different phage groups and define new ones [18], [40]. We found a remarkable diversity of this phage packaging gene in our assembled contigs. A phylogenetic analysis showed that these contigs not only recaptured several known lineages (e.g. T4-like or T7-like) but also defined many novel major branches (Figure 1).

### Recovering Complete Phage Genomes and Assigning Putative Hosts

We organized the 1148 contigs into sequence identity clusters (see Materials and Methods) to group together genomic fragments of the same or highly related phage lineages (more than 95% nucleotide identity over at least 20% overlap). It seems apparent from the examination of the contigs that the DNA from which they derive are not individual phage genomes but the concatamer that appears as an intermediate stage during the replication of most Caudovirales [41]. This has allowed us to assess genome completeness when one fosmid covered more than one complete genome in the cellular concatamer or two identical clones overlapped (Figure S1). Even though the insert size of fosmid clones (30–40 kb) limits the maximum size, two hundred and eight such complete genome representatives (henceforth referred to as CGRs) could be recovered. We have largely focused on their analyses, although the other contigs have been also used when they could provide additional information. All contigs were named in a way to reflect their origin, completeness, and sequence similarity amongst themselves (see Materials and Methods for details).

**Figure 2:**
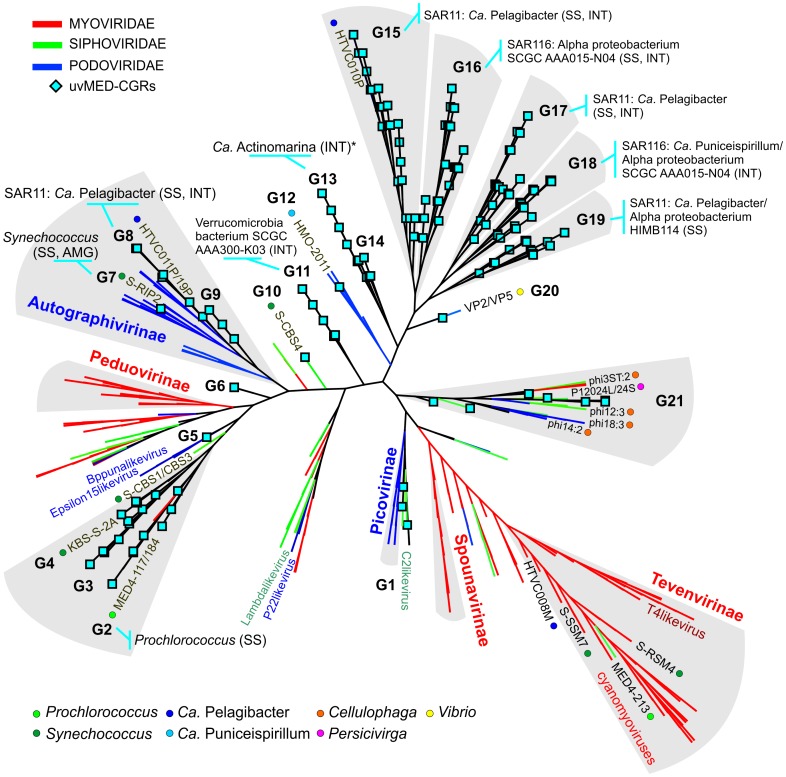
Genomic comparison of novel, complete phage genomes (CGRs) with known tailed phages. An all-vs-all comparison of several reference tailed bacteriophage genomes with the 208 CGRs identified in this study was achieved by a clustering based on a sequence similarity derived metric (see Materials and Methods for details). Branches are colored according to phage family classification (See color key top left). Branches representing unclassified phages are shown in black. The ICTV (International Committee on Taxonomy of Viruses) nomenclature of several phages is also shown for reference. In addition, color dots indicate positions of phages infecting important marine microbes (color key at the bottom). The CGRs in this study are represented by blue diamonds at the tip of the branches, and the CGR groups are highlighted in grey and labeled (G1–G21). For those groups where host prediction was possible for one or more CGRs, a taxonomic rank of the host and the organisms supporting the prediction, and the nature of the evidence supporting the assignment (*SS*: sequence similarity, *INT*: integrase/att) are shown. The asterisk (*) in G13 indicates that host prediction was performed using an incomplete genome and not a CGR.

To establish the relationships of the novel phage genomes with known phages, we performed a large-scale whole genome comparison with several reference genomes, including all marine phages. A purely genomic approach to classify phages has been proposed before and actually recapitulates several features of traditional phage classification [42]–[44]. Our slightly modified genomic approach similarly agrees well with both methods (Figure S2, S3 and S4). The whole genome comparison of the 208 CGRs shows that while some of them cluster with known phages, there are several instances of completely novel phage groups (Figure 2, Figure S5) as already hinted by the terminase phylogeny (Figure 1). Using the tree obtained, we have organized these CGRs into 21 sequence groups (G1 to G21) (Figure 2, Table 1). Within each group there was also a large degree of variation, showing protein identities typically in the range of 50–70% (see below), in effect akin to different genera of phages, i.e. within each group there was more than one phage genus. As an example, G21 groups together different genera of phages from the marine Bacteroidetes *Cellulophaga* [45] and *Persicivirga* [46] (Figure S5).

**Table 1:** General features of CGR groups and putative host assignment.

CGR group identifier	No. of CGRs in group	Average GC%	Average length (bp)	Putative host indicated for members of this group	Evidence
G1	3	35.9	37028	-	-
G2	11	36.7	38586	Cyanobacteria	*Prochlorococcus* (SS)
G3	11	49.0	41598	-	-
G4	5	49.2	40325	-	-
G5	1	35.8	40289	-	-
G6	2	54.5	34532	-	-
G7	2	46.0	44075	Cyanobacteria	*Synechococcus* (SS, AMG)
G8	7	39.6	40595	SAR11 cluster	*Ca.* Pelagibacter (SS, INT)
G9	4	54.9	42841	-	-
G10	1	53.0	43957	-	-
G11	7	45.2	38903	Verrucomicrobia	Verrucomicrobia bacterium SCGC AAA300-K03 (INT)
G12	1	56.0	41524	-	-
G13*	10	35.4	35410	Actinobacteria	*Ca.* Actinomarina minuta (INT)
G14	5	34.4	35580	-	-
G15	32	35.1	36262	SAR11 cluster	*Ca.* Pelagibacter (SS, INT)
G16	29	35.2	35537	SAR116 cluster	Alpha proteobacterium SCGC AAA015-N04 (INT)
G17	25	34.5	36042	SAR11 cluster	*Ca.* Pelagibacter (SS, INT)
G18	22	44.2	35814	Alphaproteobacteria	*Ca.* Puniceispirillum/Alpha proteobacterium SCGC AAA015-N04 (INT)
G19	20	31.9	31299	SAR11 cluster	*Ca.* Pelagibacter/Alpha proteobacterium HIMB114 (SS)
G20	1	40.4	42335	-	-
G21	11	34.5	34994	-	-

CGR (Complete Genome Representative): contig representing a complete phage genome of a cluster of highly related contigs (>95% identity and 20% coverage in nucleotide sequence). The putative host taxon assigned to one or more CGRs in a group is shown. The last column shows the evidence for host assignment in brackets next to the host name. SS: putative host inferred by the high sequence similarity to known phages; AMG: Auxiliary metabolic gene; INT: putative host inferred by an exact match of a putative phage site-specific attachment site (attP) in an integrase carrying CGR to a host tRNA (host site-specific attachment site attB). The asterisk (*) in G13 indicates that host prediction was performed using a GF (genome fragment of an incomplete phage genome) and not a CGR.

Another way to classify phages is by the host upon which they prey. Although the identification of hosts of uncultured viruses is non-trivial, phage genomes sometimes display features that divulge the identity of the host. Well known amongst such features are AMGs, metabolic genes that are frequently phage versions of host genes, e.g. photosystem genes carried by cyanophages that help boost phage replication during infection [47]–[50]. Actually, the photosystem genes (*psbA* and *psbD*), apart from unequivocally linking a phage to cyanobacteria, have been shown to discriminate not only between phages of different environments (e.g. marine or freshwater), but even different phage types (e.g. podoviruses or myoviruses) [51], [52]. In absence of such signature genes, another tell-tale feature may be simply high sequence identity to phages with known hosts, which is likely only if the phages share a common host species. For some phages, presence of CRISPR spacers in uncultured phage genomes and concordant matches in a host genome may also be used as evidence of a phage-host relationship [53]. In our case this last approach did not help, probably due to the scarcity of CRISPR systems among marine microbial genomes.

**Figure 3:**
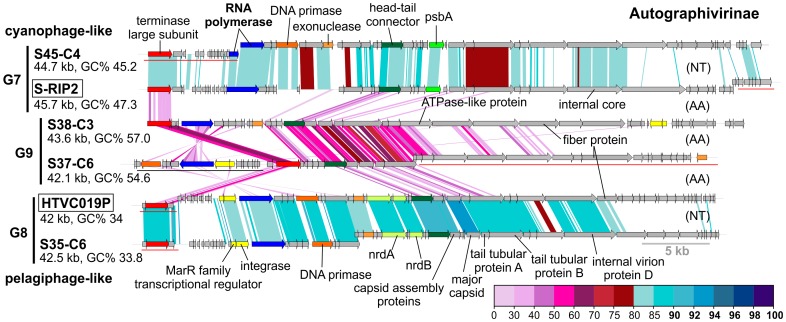
Novel Autographivirinae phages. G7, G8 and G9 belonging to the subfamily Autographivirinae are compared to each other, and to the closest related reference phage genomes (boxed). CGR names are abbreviated, e.g. S45-C4 for uvMED-CGR-U-MedDCM-OCT-S45-C4 (for the complete names see Data S1). Size and GC% of the CGR/phage genomes are also indicated. Selected genes are uniformly colored and labeled. AA (amino acids) and NT (nucleotides) labels to the right indicate if the genome comparisons were made using TBLASTX or BLASTN. A color scale for the %identity (protein or nucleotide) is shown on the bottom right side. A 5 Kb length scale is also shown (bottom right). Some gene clusters are shown displaced and underlined in the graphic indicating that they have been moved to improve comparison across all genomes.

A less explored link between phages and their hosts is related to the putative temperate nature of several phages, particularly their integration into host tRNA genes. Integration into a host genome requires that the phage carries an integrase, an excisionase and a repressor [54], [55]. Phages integrating into tRNAs carry a phage attachment site (*attP*) that is an exact match of a host tRNA gene (bacterial attachment site, *attB*). For example, the *Prochlorococcus* phage P-SS2 contains an integrase gene, and an *attP* site (53 bp), which is an exact match of 36 bp to the host tRNA (*attB*) of *Prochlorococcus* MIT9313 [56]. Along these lines, a phage carrying an integrase and a putative *attP* site identical to a host tRNA gene fragment is highly suggestive of a host-phage relationship. As proof of principle for this method, we used two cyanophage contigs from our collection identified clearly due to presence of photosystem genes (*psbA* in this case). Both these contigs also carried an integrase gene and a partial tRNA gene. Comparisons to the cyanobacterial genomes of *Prochlorococcus* and *Synechococcus* revealed that the tRNA gene fragment in both of these contigs was identical to the tRNA-Leu of *Prochlorococcus marinus* MED4 (42 bp exact match) and *Synechococcus* CC9605 (39 bp exact match), linking them to these putative hosts. Phylogenetic analysis of the *psbA* gene additionally supported this specific prediction (Figure S6 and Figure S7). Another such prediction could be made for a CGR that was >80% identical (in nucleotides) along its entire length to pelagiphage HTVC019P (Figure 3). Such high sequence identity already suggests that this CGR represents a novel pelagiphage. This CGR also carries an integrase gene and a fragment of a tRNA-Leu gene that is identical (46 bp) to the tRNA-Leu gene in *Ca.* Pelagibacter HTCC7211. It is important to emphasize that, given the high conservation of the tRNA gene among closely related species, the predictions based on this method alone are expected to provide only a broad taxonomic assignment, i.e. the phylum or class (e.g. SAR11 cluster or Verrucomicrobia). However, when supplemented with supporting evidence in the form of characteristic host genes (e.g. *psbA* for cyanophages), or high nucleotide identity to cultivated phages, it may be possible to be more specific in the predictions.

Using a combination of these approaches, applied to all the phage contigs, we were able to assign putative hosts to 527 contigs (Data S1). Several CGRs could be associated with a known host (Figure 2, Table 1, Data S1) (see below). Many of which are as yet uncultured microbes known only by their genome sequences. They represent a wide variety of important marine microbes like Cyanobacteria (*Prochlorococcus* and *Synechococcus)*, members of the SAR11 clade (*Ca.* Pelagibacter and the Alpha proteobacterium HIMB114), SAR116 representatives (*Ca.* Puniceispirillum), Verrucomicrobia and the recently described low-GC clade of marine Actinobacteria [57]. However, it is important to underscore that host-association of a single CGR in a group in no way implies that the entire group to which it belongs infects the same host. With all these caveats and after comparison of the closest known phages for each group in Figure 2, inferences regarding putative hosts could be made for 64 of the 208 CGRs. Using these host assignments and the genomic properties of the phages we classified them as follows.

### Phage Genomes Related to Known Isolates

Group G2 contains CGRs that appear to be cyanophages, likely infecting *Prochlorococcus*. They are related (>75% nucleotide identity in several regions) to the known *Prochlorococcus* phages MED4-117 and MED4-184, both dwarf myoviruses. Groups G7, G8 and G9 were closely related and actually all belong to the subfamily Autographivirinae. They all possess an RNA polymerase that is the hallmark gene of this family, among other characteristic structural and replication genes [43] (Figure 3). All RNA polymerase containing CGRs could be classified in one of these three groups. G7 contains a novel CGR that, from the *psbA* gene phylogeny (Figure S6) and similarity to *Synechococcus* phage RIP2 (>75% identity across the genome), likely preys on *Synechococcus*. Group G8 contained a CGR that could be classified as a new pelagiphage (infecting SAR11) by both sequence similarity (>75% nucleotide identity along the entire genome) to HTVC019P [32] and the integrase/att relationship. CGRs in group G9 are novel phage genomes for which no host assignment was possible.

**Figure 4:**
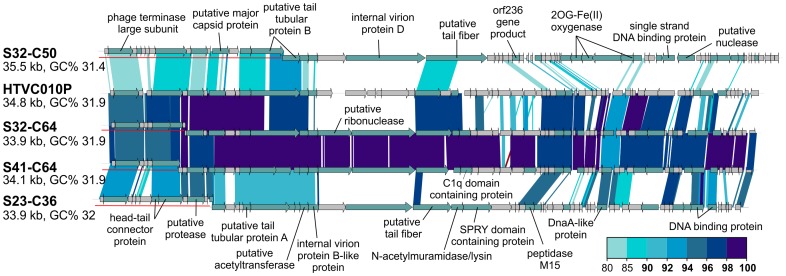
Novel Mediterranean *Ca.* Pelagibacter phages. Four CGRs representing novel *Ca.* Pelagibacter phages are shown in comparison to the cultivated pelagiphage HTVC010P. CGR names have been abbreviated as in Figure 3. All comparisons were done at the nucleotide level. A color scale for the % identity is shown on the bottom right. Representation of the contigs is as in Figure 3.

Of the 31 CGRs in group G15, nine seem to be related to the recently cultured Pelagibacter phage, HTVC010P, which was shown to be the most abundant phage in the oceans [32]. These CGRs shared high nucleotide identities (>80%) in large regions with HTVC010P, suggesting they are also pelagiphages. In particular, two of these CGRs are highly similar along their entire lengths to HTVC010P, effectively making them Mediterranean variants of this phage, which was isolated from Bermuda (Hydrostation S) (Figure 4). Additionally, several of these new phage genomes were linked to the SAR11 cluster by the integrase/att relationship (Table 1, Data S1).

### Novel Phage Groups

We defined several distinct groups of phages for which there are no known related genomes available. However, it was still possible to predict hosts for several CGRs in these novel phage groups. For example, one of the seven CGRs in group G11 could be linked to Verrucomicrobia using evidence from integrase/att identity to the single-cell amplified genome (SAG) SCGC AAA300-K03 [14] recently described as belonging to this phylum. The GC content of this phage genome (43.8%) also matches very well the cellular genome GC content (42.3%). To our knowledge this is the first report of a marine Verrucomicrobia phage.

G17 and G19 contained CGRs that were putative pelagiphages unlike any others known before. There is evidence for them infecting SAR11 cluster microbes from both integrase/att relationship and small regions of high nucleotide identity with HTVC010P. In addition, some of the CGRs in G19 were nearly fully syntenic to a prophage locus in the genome of the SAR11 alpha proteobacterium HIMB114, albeit at a protein sequence identity in the range of 40–50% (Figure S8).

Along the same lines, several of the CGRs from the group G16 (Table 1, Figure 2) could prey upon SAR116. The first SAR116 phage (HMO-2011) has been recently described [58]. However, these CGRs are unrelated to HMO-2011, which is related to group G12 instead (Figure 2). Only the integrase/att relationship connected these CGRs of group G16 to *Candidatus* Puniceispirillum marinum [59] and other uncultured SAR116 representatives [14].

One phage genomic fragment (not a CGR) could be putatively assigned as an actinobacterial phage, the most likely host being *Ca.* Actinomarina minuta, the smallest free-living microbial cells described so far [57]. The fragment carries an integrase and also a 43 bp *att* site that is 100% identical to tRNA-Val of the putative host genome. This match is so specific that the att site sequence only retrieves *Ca.* Actinomarina minuta sequences from the complete GenBank collection. In addition, a WhiB transcriptional regulator found only in Actinobacteria, was also found in this phage fragment. This gene has been found previously in mycobacterial phages (e.g. TM4), where it has been shown to have a growth inhibitory and a super-exclusion effect [60]. This phage genomic fragment appeared most closely related by sequence (Figure 2) to the G13 group of CGRs to which no other host could be assigned.

### Abundance of CGRs in the Marine Environment

**Figure 5:**
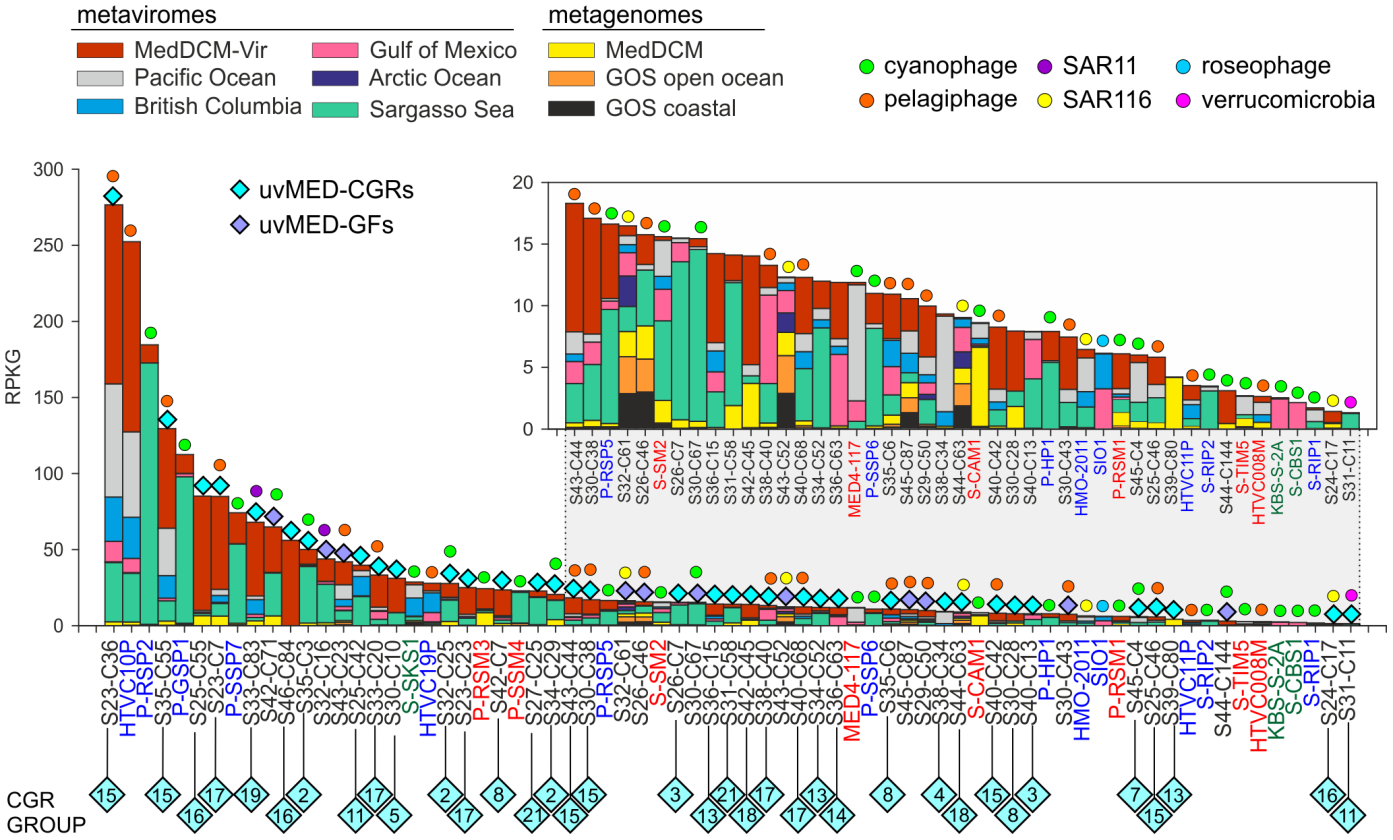
Comparative fragment recruitment of CGRs, GFs contigs (genome fragments of incomplete phage genomes) and representative cultivated phage genomes. Number of reads recruited by each, expressed as RPKG (Reads per Kb per Gb) from several metaviromes and metagenomes (color coded). Only hits that had >95% identity, minimum length of 50 bp and e-value<1e-5 were considered in this analysis. The names of the fosmid contigs are abbreviated as before. The reference genome names are highlighted in color according to their family (*blue*, Podoviridae; *green*, Siphoviridae and *red*, Myoviridae). Blue diamonds on top of the bars mark the contigs identified in this study with different colors (*cyan*, CGRs; *purple*, GFs) and the predicted (or known) host is also indicated when possible. Inset is a magnified view of the lesser recruiting fragments.

An essential question is how relevant are the phages represented by our CGRs in a DCM phage population. To this end, a different DCM sample from the same location (and retrieved four years later) has been processed to generate a metavirome (MedDCM-Vir). The DNA from the viral fraction in the sample was amplified by MDA and sequenced by Illumina to provide nearly 18 Gb of sequence data. We used this metavirome, along with several others [21], [22], and some representative metagenomes, to assess relative abundance of known marine phages (133 reference genomes) and the CGRs. The most abundantly recruiting genomes are shown in Figure 5 and Figure S9. As expected, recruitment from metagenomes is much less than from metaviromes, reasserting the viral nature of CGRs. Among the top recruiting genomes there is a large representation of the CGRs, with only a few cultivated *Ca.* Pelagibacter and *Prochlorococcus* phages reaching comparable values. Although most CGRs recruited more in their habitat of origin (MedDCM-Vir) they also recruited very well in other datasets, such as the Sargasso Sea. Reciprocally, several phages isolated from the Sargasso (e.g. P-SSP2, P-GSP1 and P-SSP7), not only recruited a high number of reads from the Sargasso Sea metavirome, but also from the MedDCM-Vir.

How much of the viral diversity at the DCM was recovered in our fosmids? To answer this question, we have used very relaxed criteria for recruitment (BLASTN, minimum alignment length 50 bp and e-value 0.01). As a control, we used multiple genomes of several abundant DCM microbes (*Prochlorococcus*, *Synechococcus*, *Ca.* Pelagibacter, SAR86, Group II Euryarchaeota, adding up to a total of 75 Mb sequence data) to recruit reads from the metavirome MedDCM-Vir. Only 0.14% of reads could be matched indicating a negligible contamination with cellular DNA. On the other hand, the 1148 phage contigs described here recruited about 1.54% of all the reads of the MedDCM-Vir, an order of magnitude more, but still suggesting they represent a small minority in the Mediterranean DCM virome. The 133 reference genomes recruited even less (only about 0.36%). It has been recently suggested, using microscopic techniques, that natural marine viral populations may be dominated (up to 92%) by non-tailed phages [61], providing a potential explanation for such low recruitment levels.

It is important to underscore here that the metaviromes are always amplified by MDA. There is evidence that MDA acts much more efficiently with single stranded DNA so that extant metaviromes could be over representing ssDNA viruses [19], [20] and are consequently biased against dsDNA genomes. However, even the recently described 608 genomes of marine, circular ssDNA viruses [26] recruited only 1.5% of the MedDCM-Vir reads. Such results are not restricted to the Mediterranean DCM metavirome, as they recruited similarly low levels from the Sargasso metavirome (0.89%) [21], and nearly nothing from the Pacific Ocean Virome [22]. Therefore, it appears that the vast majority of the marine virome sequence space is as yet unsampled.

### Patterns in Concurrent Phage Diversity and Clues about Biogeography

With this large collection of complete phage genomes from the same place and time, it becomes possible to examine concurrent diversity, and patterns of variability, that have traditionally been analyzed by repeated and independent phage isolation in culture. Firstly, we have found several examples of nearly identical phage genomes. Using very restrictive similarity criteria (95% nucleotide identity over 95% overlap) we identified 519 contigs (out of the total of 1148) that clustered in groups of 2 to 22 members. Analysis of these highly similar clusters revealed several examples of nearly identical phage genomes with minor differences only, clearly showing that there are numerous recently diverged concurrent variants. For example, cluster C12B (Figure S10) contains nine contigs that were >98% identical over the overlapping regions. In this comparison, some contigs are nearly identical with only minor indels, such as contigs 6 and 7 in Figure S10. However, other contigs/regions diverged much more (similarity down to 75–80%, for example contigs 7 and 8). This is reminiscent of the flexible genomic islands of the prokaryotic genomes [62] and had previously being shown for cultivated phages. In a number of published studies [27], [53], tail proteins and other host recognition structures have been described as highly variable. This phenomenon was attributed to diversity of host recognition specificity among different phage lineages. However, the variations that we have found in the closely related genomes, although including structural host recognition features, do not appear to be restricted to any specific functional role. For example, internal virion proteins, terminases and capsid proteins were all observed within variable regions.

Another frequent pattern is the presence of a hybrid architecture in which large divergent regions are present together with regions of high identity. An example is shown in Figure S11. Such genomes clearly belong to phages infecting a common host that have exchanged genomic fragments during a mixed infection. Moreover, given the identical nature of several of these regions, it does appear that these exchanges are recent events. Similar results have been obtained by comparing cultured phage isolates. For example, the study of several isolated staphylococcal phages strongly suggested the exchange of large segments of genomes among them [63]. Whether or not such recombinations are facilitated by the presence of linker regions [64] or are random rearrangements followed by selection for function has not been established. The sheer amount of phage infections occurring in the marine habitat at any given time [1], [2] makes it likely that any of these events are feasible.

Given the high sequence identities found between the Mediterranean pelagiphages and the first pelagiphage HTVC010P isolated from the Sargasso Sea (Figure 4), we searched for more such examples in our contig collection. Identical phage sequences have been found before in geographically distant marine samples, but these were based on small genomic fragments (200–600 bp) [65], [66]. We found two cyanophage contigs from our collection that were >97% identical along their entire lengths to cyanophages isolated as far as the Pacific Ocean (Figure S12). Both of these contigs were nearly 40 Kb long (nearly complete fosmids) and originate from myoviruses that are >170 Kb long. These are remarkable examples of global distribution of viruses that suggest a rapid global phage circulation, likely along with oceanic currents.

## Discussion

It has been clear for some time that culture, although instrumental in the development of Microbiology, cannot provide an adequate representation of the real diversity of prokaryotic microbes and their phages in a sensible timeframe. New technologies based on high-throughput sequencing and direct nucleic acid retrieval from communities or single cells provide critical short-cuts for advancing in the discovery of the cellular microbes. However, an equivalent short-cut for the phage sequence space has been missing. Phage isolation and sequencing is very important in studying the natural diversity of phage populations, yet it is tightly constrained by the burden of obtaining host cultures. These limitations are not only relevant for the study of biodiversity alone. The population genomics of prokaryotic microbes and their phages, i.e. their evolution and microdiversity, suffer from similar handicaps. Here we have provided the largest collection of concurrent phage genomes ever described for any habitat so far by using metagenomic fosmids. This opens a route towards phage population genomics that can be based on complete genomes, rather than small genomic fragments. It appears to be the simplest and most effective high-throughput method to obtain complete phage genomes from a natural habitat yet. In addition, we have been able to assign putative hosts to many by using sequence based criteria that appear quite reliable and could prove instrumental as the field of metaviromics evolves further.

On the other hand, it is quite obvious that we have only retrieved a small fraction of the full diversity of phages living in the habitat of choice (the Mediterranean DCM). First of all our method is limited by the size of fosmid clones so that large viral genomes could not be retrieved. We could have tried to use overlapping fosmids but they would probably lead to unreliable chimeric assemblies. The genuine examples of mosaicism detected here, make artifactual assemblies from metaviromes a possibility that needs to be considered. For now we decided to focus mostly on the *bona fide* complete genomes (CGRs). One possible way to bypass the size limitation would be to use larger insert vectors such as BACs [67], or apply long-read sequencing directly to the samples when it is available [68]. Another obvious limitation of our method is that only replicating viruses, and apparently, those using the concatamer mode of replication, have been captured. Although it appears to narrow the window of the kinds of phages detected, it provides a confirmation of their active role in the ecology of the environment. It is possible that some phage particles are just remnants of past lytic events [1], which are not relevant for the current habitat ecosystem functioning despite their presence in the metavirome. Those are excluded in our methodology. Finally, both single stranded DNA or RNA phages [69] are obviously omitted from detection by our technique. As mentioned before, traditional metaviromes might also be highly biased [19], [20], [70], and in this sense both methods might be complementary. In spite of all these caveats, we nearly tripled the number of marine phage genomes.

Given the recovery of nearly identical genomic fragments across the globe, it is already evident that there is very weak (if any) phage biogeography in temperate and tropical latitudes. Therefore, an in depth study of a single location can contribute enormously to our knowledge of phage biodiversity. Furthermore, coming from a single sample, we have shed light into the dynamics of genome change in concurrent phages. Using phage contigs highly related to the globally distributed pelagiphage HTVC010P, remarkable sequence conservation and variation patterns were discernible. There are aspects that are reminiscent of similar phenomena in prokaryotes [71]–[73], such as the flexible genomic islands, i.e. the presence of several concurrent lineages that differ only in small genomic regions. Some of these regions are probably involved in host specificity at the level of clonal lineages [5], [27]. However, some unexpected genes were subjected to high microdiversity (capsid protein and terminases), the reasons for which are for the moment obscure. Capsid proteins could be involved in host recognition but it is not likely that the terminase has any connection with such specificity. In addition, swapping of genome fragments amongst phage lineages appears to be a central theme in phage evolution. Overall there seems to be more creativity in concurrent, highly identical (over 95%), phage genomes compared to cellular genomes, that sometimes involves the replacement of large genomic segments, likely by recombination with distant lineages that share the same host. Similar phenomena had been detected before in cultivated phages [63]. Not being strictly fitness constrained as the cellular compartment, phages might embark onto more adventurous evolutionary trajectories. Actually, there is little doubt that phages may represent a significant part of the prokaryotic pan-genome [74] that could outsource risky, but highly innovative, evolutionary paths to their accompanying phage populations.

The availability of large numbers of closely related genomes and the discernible patterns in their diversity and distribution increases our appreciation towards the enormous variety that exists, much of which was only partially captured before by isolated phage genomes. Importantly, it opens up a view of the phage world where instead of observing phage genomes as discrete entities, we can begin to look upon them as vast, constantly churning global continuums.

## Materials and Methods

### Sample Details, Fosmid Sequencing, Assembly and Phage Contig Identification

The sample from which the fosmid library was constructed was taken on October 15, 2007 from the DCM (50 m depth) off the coast of Alicante, Spain (38°4′6.64″N 0°13′55.18″W) with a Niskin bottle. The sample was filtered through 5 µm polycarbonate and 0.22 µm Sterivex filters. DNA from 0.22 µm filters was used to create a fosmid library of ∼13000 clones. A 454 metagenome from the same filter, and results of sequencing of ∼1000 fosmids have been described previously [10]. For this work, DNA from ∼6000 metagenomic fosmids was extracted and pooled in 24 batches, with ∼250 fosmids in each batch. These were sequenced using Illumina PE 300 bp reads in a single lane (∼175× coverage for each fosmid). Each batch was assembled independently using Velvet [75] (k = 51). Several criteria were employed to identify phage genomic fragments, for example, multiple hits to all known phages, presence of key phage genes using Phage Orthologous Groups [37], availability of multiple related fragments, and manual examination of each contig. POGs are clusters of orthologous genes from bacteriophages that can be used to identify viral genes and a virus quotient (VQ) quantifies the phage specificity of each gene (the closer it is to 1, more phage specific the gene is). The VQ profile of the POGs of selected MedDCM contigs was very similar to the one obtained for the known phage genomes (majority of the POGs with VQ equal to 1), suggesting that those contigs indeed represent true phage genome fragments. A total of 1148 (lengths ranging from 5–48 kb) contigs were finally selected for the final analysis. The presence of the vector sequence (ranging to 16–67 bp) on both sides of 139 assembled contigs indicated that these contigs represented the complete fosmid sequence. The lengths of the majority of the complete fosmids were between 30–40 kb. Genes were predicted using prodigal [76], and annotated using BLAST against the NR database, Pfam [77], COGs [78], TIGRfams and POGs [37]. All complete genome representatives were manually examined and annotated using the HHpred server [79]. All contigs were named according to the nomenclature described below.

### Metavirome Sampling, Construction, Sequencing and Assembly

Seawater (20 L) collected from the DCM of the Mediterranean Sea (65 m deep) on August 29^th^, 2011, was filtered through a 0.2 µm filter (Millipore GVWP2932A). Subsequently, phages were concentrated using tangential flow filtration (TFF) with a 30 kD polyethersulfone membrane from Vivaflow (VF20P2). The resulting phage concentrate was ultracentrifuged (Optima XL 1000K Ultracentrifuge, Beckman) for 1 h at 4°C using a Type 70 Ti rotor (Beckman) at 30,000 rpm (92,600 g). The pellet was resuspended in 1 mL of the seawater supernatant and treated with 2.5 units DNase I at 37°C for 1 hr, and 70°C for 10 min to remove bacterial DNA. The phages were then lysed in 0.50 mg/mL Proteinase K and 1.0% SDS at 56°C for 1 h followed by two rounds of phenol/chloroform/isoamyl alcohol extraction. The aqueous phase was then chloroform/isoamyl alcohol extracted and ethanol precipitated and resuspended in sterile water. DNA quantity and quality was determined using gel electrophoresis and Picogreen. Multiple amplification displacement (Illustra GenomiPhi V2 DNA Amplification Kit, GE Healthcare) was performed using ca. 30 ng of DNA for each of five reactions. The resulting DNA (ca. 5 µg) was sequenced in one third of an Illumina lane, yielding approximately 18 Gb of sequenced data (paired end reads, 300 bp insert size) with a total of ∼180 million reads.

### Recovery of Complete Phage Genomes

An all-vs-all comparison, using BLASTN [80] was performed for all contigs. Only >95% identical hits and with lengths >50 bp were retained. Overlapping hits, if any, were merged together using the mergeBed program in the BEDtools package [81]. The total length of these hits was then used to compute percentage coverage of the contig length. All pairs of contigs selected satisfying the coverage criteria (of 20% in the first round of clustering and 95% in the second round) were visualized in Cytoscape as a connected network [82]. Groups of connected contigs in these networks were considered as valid clusters. The 1148 contigs were clustered first using a criterion of >20% coverage but with very high nucleotide sequence identity (>95%). 117 clusters (containing 914 contigs) were obtained, and 236 contigs remained unclustered. In the next step, the contigs in each cluster were clustered at an even stricter criterion of at >95% coverage and >95% nucleotide identity to identify nearly identical contigs. Further examination of the 102 subclusters obtained after this second step, allowed us to identify 208 complete phage genomes indicated by the circular-like organization of two or more contigs of a cluster. Similarly, end redundancy in contigs that were unclustered was used to identify complete genome representatives.

### Nomenclature of Viral Contigs

As described above, an all-vs-all nucleotide comparison was used first to cluster all viral contigs using cut-off of 95% sequence identity and 20% coverage. Contig clusters formed in this step were named given a cluster number (e.g. C1, C2 etc). Unclustered contigs were tagged with a “U”, for “unclustered”. In the second round of clustering, we used the same sequence identity (95%) but a higher cut-off to coverage (95%) to identify the most highly related and syntenic contigs within each cluster. At this stage, if multiple clusters were obtained within a single cluster (say C1), they were tagged alphabetically, e.g C1A, C1B, C1C etc. Contigs within a cluster (C1), but not part of any further subclusters were not tagged again. All clusters (both clusters and subclusters) were examined manually for completeness. For example, if a complete genome representative (CGR) was identifiable in subcluster C1A, it was tagged as a CGR-C1A. If a CGR was identifiable in a cluster, it was tagged as CGR-C1. If a CGR was found in a cluster, all other contigs that were not identified as CGRs, were tagged as CGF (complete genome fragment). The naming scheme is described in detail below.

### Nomenclature Scheme for Uncultured Viruses Used in This Work

Following the suggestions made for the nomenclature of viruses, we have used the following procedure for the nomenclature of uncultured viruses described in this work: (1) uv - uncultured virus (2) MED - three letter abbreviation in capitals indicating origin of the sample (3) CGR/CGF/GF - field indicating if the contig refers to a complete genome, a fragment of a complete genome, or just a genomic fragment. CGR (complete genome representative) is a contig that is assumed to be a complete phage genome. There may be more than one CGR in a cluster. CGF (complete genome fragment) is a contig that cannot be inferred to represent a complete phage genome, but is part of a cluster that contains a CGR). GF (genomic fragment) is similar to a CGF but without any CGR. Such GF contigs can have an extra name field (see below) indicating they are unclustered (U). (4) C1/C1A/C1B/U - indicates the clustering status of the contig. (5) Field containing the contig identifier, e.g. MedDCM-OCT-S14-C437. An example of a complete identifier is uvMED-CGR-C1-MedDCM-OCT-S17-C19, enabling quick identification of several key features of a phage genome/contig.

### Genomic Comparison of All Phages

Several well-classified reference phage genomes, identified using the ICTV classification (http://www.ictvonline.org) were downloaded from NCBI. In addition, all known marine phage genomes were included in the comparison. Each genome was compared to another using TBLASTX [80] using the BLOSUM45 matrix. A hit was considered significant if it had >30% sequence identity, a minimum length of 30 aa and an e-value of at least 0.01. The bit score of all such selected hits in a comparison was summed up to give a comparison score for a pair of genomes. Closely related genomes get higher comparison scores. To normalize for different genome sizes each phage genome was also compared to itself to obtain a self-score. The Dice coefficient, which is a similarity metric ranging from 0 to 1, was computed as follows, *Dice* = (2*AB)/(AA+BB), where AB is the comparison score of phage A with phage B, AA and BB are the comparison scores of phages A and B with themselves respectively. This metric was transformed to a dissimilarity metric by subtracting it from one. A neighbor joining tree was constructed from the complete distance matrix using the PHYLIP package [83].

Separate initial comparisons were run for well classified podoviruses, myoviruses and siphoviruses (classification obtained from http://www.ictvonline.org) to examine the validity of the approach (See Figure S2, Figure S3 and Figure S4). The tree shown in Figure 2 was created using a comparison of all reference phages and with the complete genome representatives (208 CGRs) identified in this study. In the comparison of all tailed phages to each other (Figure 2), several well described phage groups are separable, e.g. Autographivirinae, Tevenvirinae, Spounavirinae etc.

### Phylogenetic Analysis

For the terminase tree, Pfam domains, COGs, POGs, TIGRfams were searched using hmmsearch program in the HMMER3 package [84] (evalue 1e-5), in addition to NCBI BLAST [80] to identify large subunit terminase sequences in the entire dataset. 401 unique sequences were identified in the contigs. In addition, 125 reference sequences and 105 terminase sequences from marine phages were included. A total of 631 terminase sequences were used for the alignment. For the phylogenetic trees of photosystem genes *psbA* and *psbD*, protein sequences were extracted from the annotated metagenomic fosmids and compared to NCBI NR database using BLASTP to recover additional sequences. Several previously described sequences were also used. All alignments were created using Muscle [85], manually inspected and trimmed as necessary, and maximum likelihood trees were constructed using the program FastTree2 [86] using a JTT+CAT model and an estimation of the gamma parameter. Bootstrapping was performed using the Seqboot program in the PHYLIP package [83].

### Comparative Fragment Recruitment

We used both metagenomes (MedDCM [10], Global Ocean Sampling [11]) and metavirome datasets from the Sargasso Sea, British Columbia coastal waters, Gulf of Mexico, Arctic Ocean [21] and the Pacific Ocean [22]. In addition, the metavirome (MedDCM-Vir) constructed in this study was also used. For depicting comparative recruitment across metaviromes and metagenomes (as shown in Figure 5), a hit was considered if it was at least 50 bp long, had an e-value of less than 1e-5 and more than 95% identity. The number of hits to each phage contig was divided by the length of the contig (in kb) and also by the size of the database (number of reads recruited per kb of contig/size of the database in Gb), which provides a normalized measure to compare recruitments by differently sized contigs versus several metagenomes. This measure is abbreviated as RPKG (Reads per Kb per Gb).

### Accession Numbers

All 1148 contigs assembled in this study have been submitted to DDBJ and are available using the accession numbers AP013358-AP014505. The metavirome has been deposited in NCBI SRA with the Bioproject number PRJNA210529.

## Supporting Information

Data S1 (**A**) Contig information. List of all the identifiers for all contigs, their clusters, groups and predicted host (if any). (**B**) CGR groups. Information on CGRs in each group, most closely related reference phage genomes, predicted hosts (if any) and the evidences used for host prediction. (**C**) Integrase-att. List of integrase and att site containing contigs that match an att site in a host genome. The size and location of the match both in the contig and the host, host genome name, NCBI GenBank accession number, and additional evidence linking the contig to the host. (**D**) Reference genomes. List of all complete phage genomes used in this work.
(XLSX)

Figure S1 Recovering complete genomes from phage genome concatamers. (**A**) Schematic representation of phage replication via a concatamer formation in a bacterial cell, leading to natural amplification of phage genomic material. (**B**) Inferring complete genomes cloned in fosmids. A schematic representation of the phage genome concatamer is shown and boundaries of each genome are indicated by a vertical bar. Two methods for examining a fosmid for presence of a complete phage genome are shown, one by checking for identical repeats at end of the fosmid, and the other, by examining relative gene order in nearly identical phage genomes.
(PDF)

Figure S2 Genomic comparisons of Podoviruses. The two main subfamilies, Autographivirinae and Picovirinae indicated in bold. Phages belonging to the same genus and clustering together are labeled e.g. P60likevirus, T7likevirus. Some unclassified podoviruses are also shown (labeled as “others” in the phage name label). Classification details were obtained from http://www.ictvonline.org.
(PDF)

Figure S3 Genomic comparisons of Myoviruses. The three main subfamilies, Tevenvirinae, Spounavirinae and Peduovirinae are indicated in bold. Phages belonging to the same genus are labeled by the Genus name, e.g. Hpunalikevirus, Phikzlikevirus etc. Classification details were obtained from http://www.ictvonline.org.
(PDF)

Figure S4 Genomic comparisons of Siphoviruses. Phages belonging to the same genus are labeled by the Genus name, e.g. Hpunalikevirus, Phikzlikevirus etc. Classification details were obtained from http://www.ictvonline.org.
(PDF)

Figure S5 Genomic comparison of novel, complete phage genomes (CGRs), with known tailed phages. This figure is similar to Fig. 2 in the main text but contains genus names of several reference phages. All names were obtained from the ICTV classification (http://www.ictvonline.org).
(PDF)

Figure S6 Phylogenetic tree of psbA gene sequences. psbA sequences obtained from cyanobacteria, cultivated cyanophages and fosmid cyanophage contigs are shown in this tree. Sequence names are color coded as follows: *Prochlorococcus*, dark green; *Synechococcus*, dark blue; *Prochlorococcus* cyanophages, light green; *Synechococcus* cyanophages, light blue; uvMED fosmid contigs, red. Each sequence from GenBank is labeled as follows: accession number_ marine(MA)/estuary(ES)/freshwater(FW)_ Phage(Ph)/bacteria(Ba)_ GenBank description. Bootstrap values of >50% are shown as black circles at each node. Host name and phage morphology is indicated on the right for some clusters.
(PDF)

Figure S7 Phylogenetic tree of psbD gene sequences. Several psbD sequences obtained from cyanobacteria, cultivated cyanophages and the uvMED fosmid contigs are shown in this tree. Sequence names are color coded as follows, *Prochlorococcus*, dark green; *Synechococcus*, dark blue; *Prochlorococcus* cyanophages, light green; *Synechococcus* cyanophages, light blue; uvMED fosmid contigs, red. Each sequence from GenBank is labeled as follows: accession number_ marine(MA)/estuary(ES)/freshwater(FW)_ Phage(Ph)/bacteria(Ba)_ GenBank description. Bootstrap values of >50% are shown as black circles at each node. Clusters of sequences are labeled according to their origin and phage morphology wherever applicable.
(PDF)

Figure S8 Putative SAR11 CGRs. BLASTN and TBLASTX comparisons of CGRs of group G19 versus. (**A**) the prophage locus in alpha proteobacterium HIMB114. (**B**) *Ca.* Pelagibacter phage HTVC010P. A color key for the %identities is also shown.
(PDF)

Figure S9 Comparative fragment recruitment of cultivated phage genomes. Number of reads recruited by each, expressed as RPKG (Reads per Kb per Gb) from several metaviromes and metagenomes (color coded). Only hits that had >95% identity, minimum length of 50 bp and e-value<1e-5 were considered in this analysis. A magnified view for the less recruiting phages is also shown below.
(PDF)

Figure S10 Nearly identical, concurrent phage contigs. A nucleotide comparison of several highly related contigs is shown. Color key for the %identity is shown in the top right corner. All these contigs were clustered together in the cluster C12B. Contigs are labeled by a number (1, 2, 3 etc.) and the full contig names are given below. Selected genes are labeled and colored uniformly.
(PDF)

Figure S11 Concurrent, hybrid phage contigs. A nucleotide comparison of several highly related contigs is shown. Color key for the %identity is shown in the bottom right corner. All these contigs were clustered together in the cluster C10. Contigs are labeled by a number (1, 2, 3 etc.) and the full contig names are given below, along with size and GC%. Selected genes are labeled.
(PDF)

Figure S12 Mediterranean phage contigs identical to cultivated phages isolated from distant geographical locations. Three fosmid contigs are shown, each compared to the genome of a phage that was not isolated from the Mediterranean. All phage genomes and contigs are labeled and their size and GC% is indicated. A color key for the %identity of the alignments is shown in the bottom right corner. Three locations are marked in the world map (blue circle: isolation of *Synechococcus* phage S-CAM1, orange circle: isolation of *Ca.* Pelagibacter phage HTVC010P, and blue diamond: Mediterranean. Location information for the *Synechococcus* phage metaG-MbCM1 was not available. Some gene clusters are shown displaced and underlined in the graphic indicating that they have been moved to improve comparison across all genomes. The map shown in the figure was obtained from www.naturalearthdata.com.
(PDF)
