# Supplementary figures and images for: Expanding the Marine Virosphere Using Metagenomics
Source: PLoS Genet. 2013 Dec 12;9(12):e1003987. doi: 10.1371/journal.pgen.1003987 (PMC3861242; doi:10.1371/journal.pgen.1003987)

A

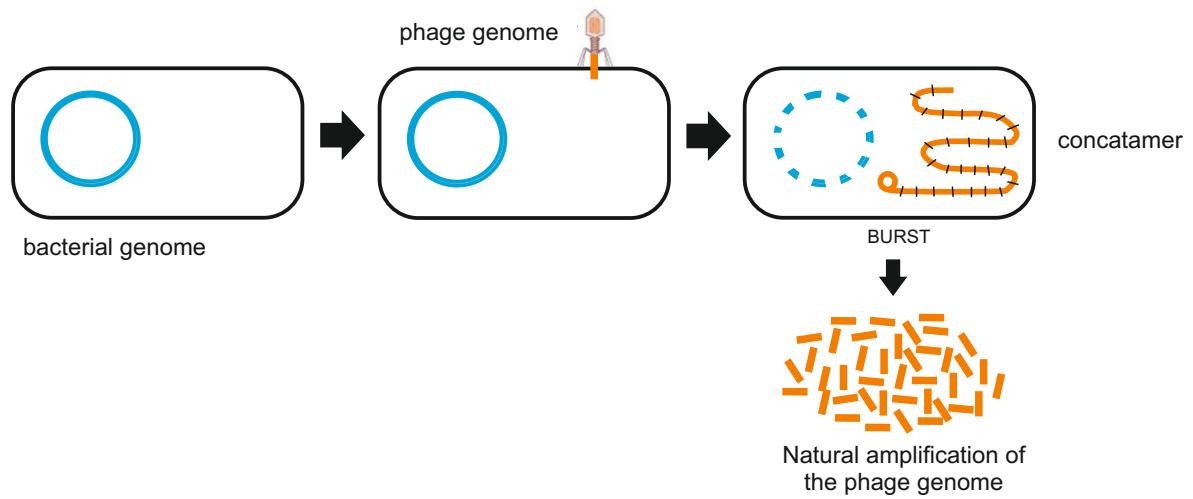

**B**

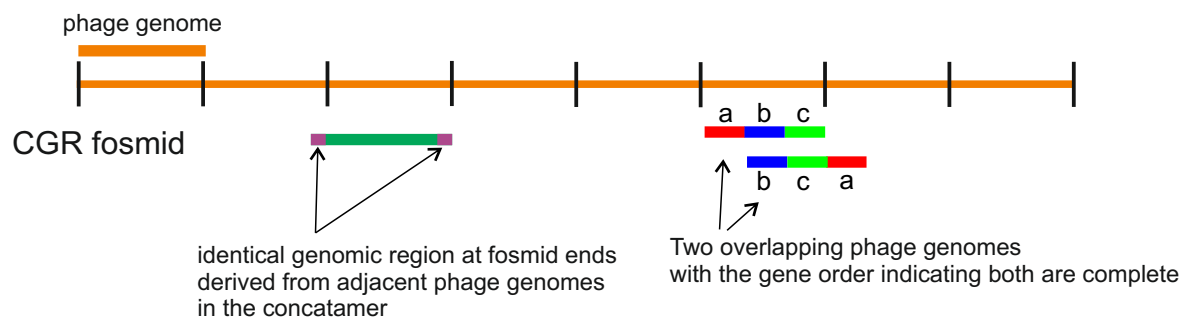

Supplement: Figure S1 — Recovering complete genomes from phage genome concatamers. (A) Schematic representation of phage replication via a concatamer formation in a bacterial cell, leading to natural amplification of phage genomic material. (B) Inferring complete genomes cloned in fosmids. A schematic representation of the phage genome concatamer is shown and boundaries of each genome are indicated by a vertical bar. Two methods for examining a fosmid for presence of a complete phage genome are shown, one by checking for identical repeats at end of the fosmid, and the other, by examining relative gene order in nearly identical phage genomes. (PDF) [file pgen.1003987.s002.pdf]

## Podoviruses

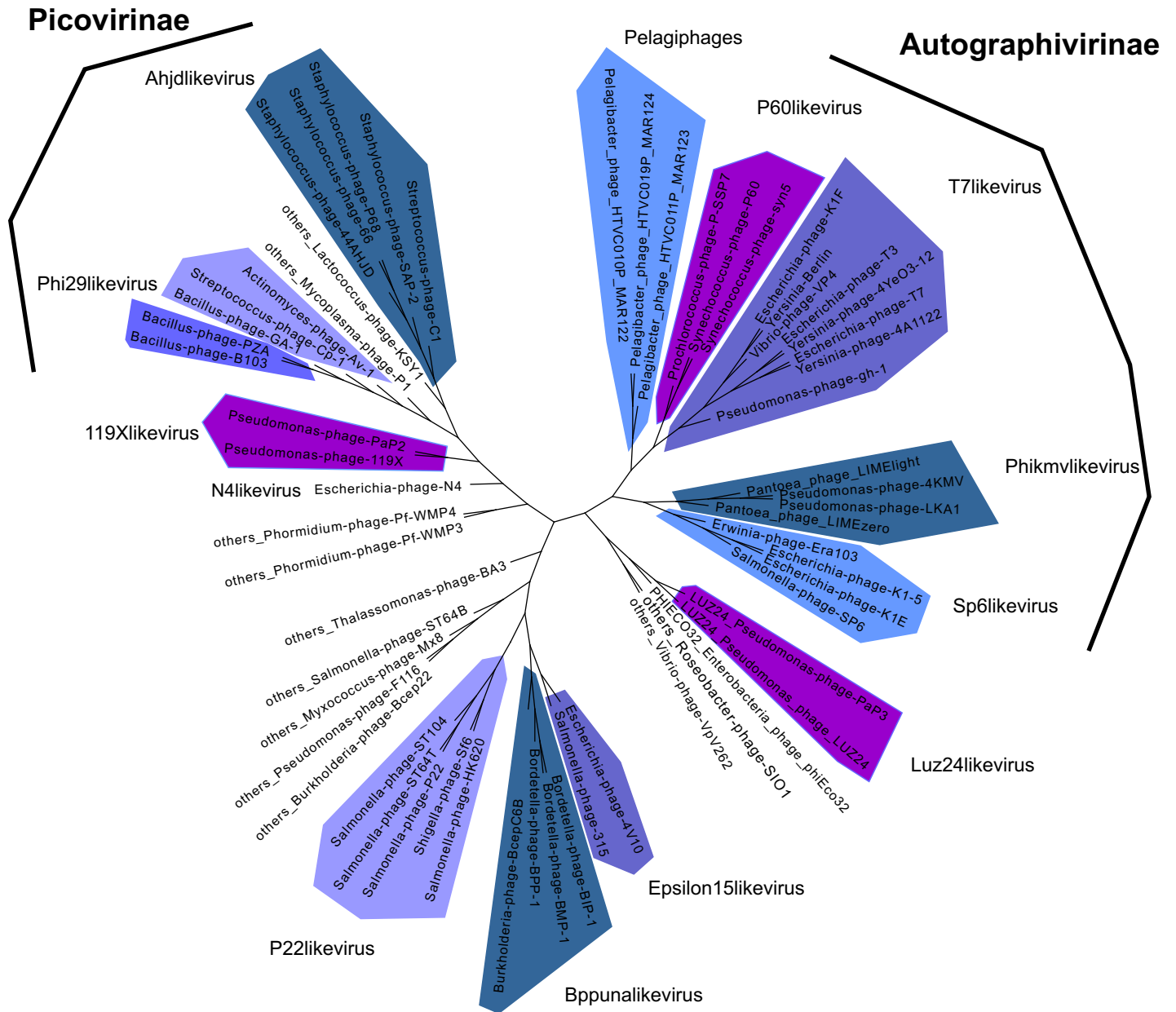

Supplement: Figure S2 — Genomic comparisons of Podoviruses. The two main subfamilies, Autographivirinae and Picovirinae indicated in bold. Phages belonging to the same genus and clustering together are labeled e.g. P60likevirus, T7likevirus. Some unclassified podoviruses are also shown (labeled as “others” in the phage name label). Classification details were obtained from http://www.ictvonline.org. (PDF) [file pgen.1003987.s003.pdf]

## Siphoviruses

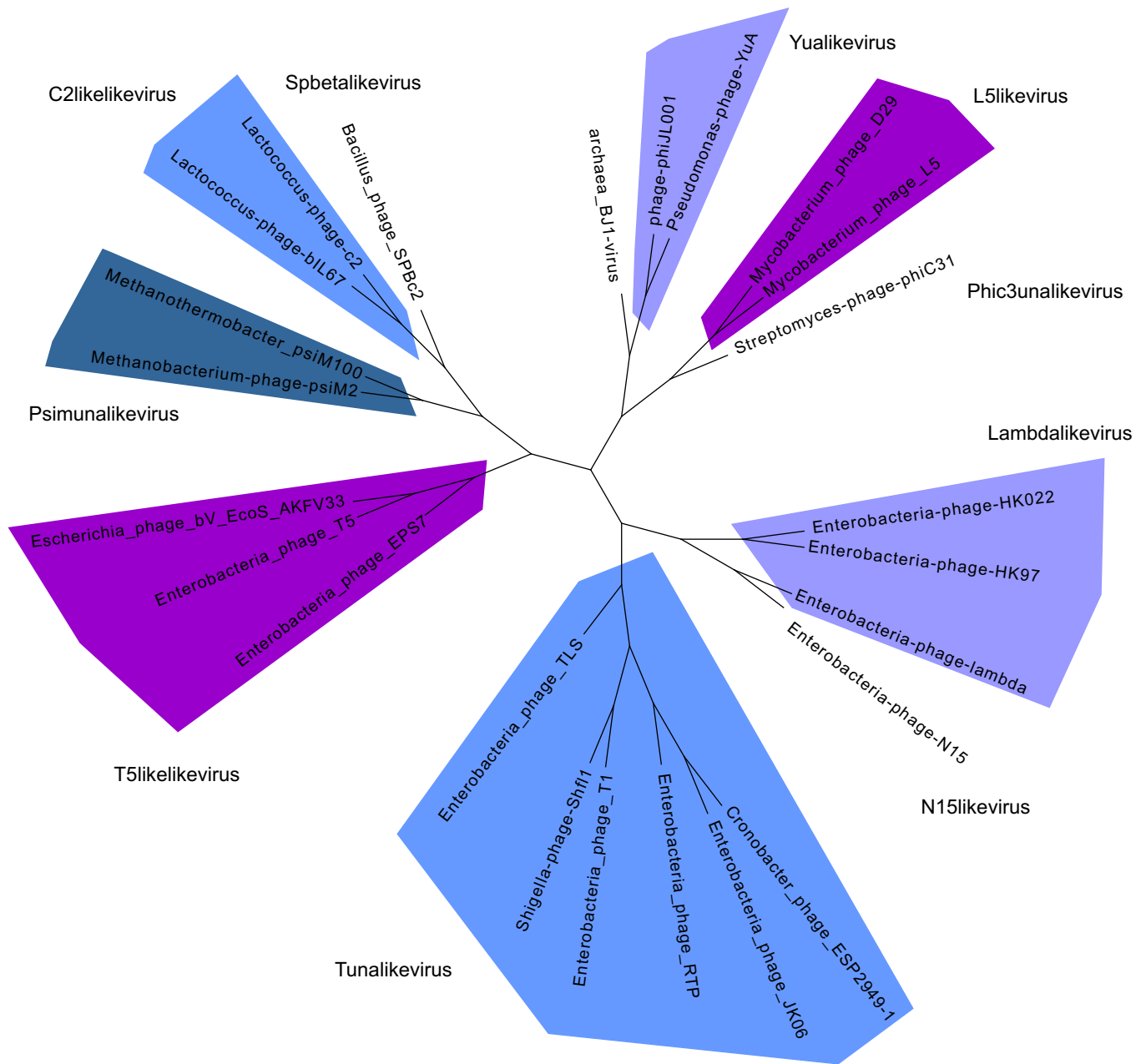

Supplement: Figure S4 — Genomic comparisons of Siphoviruses. Phages belonging to the same genus are labeled by the Genus name, e.g. Hpunalikevirus, Phikzlikevirus etc. Classification details were obtained from http://www.ictvonline.org. (PDF) [file pgen.1003987.s005.pdf]

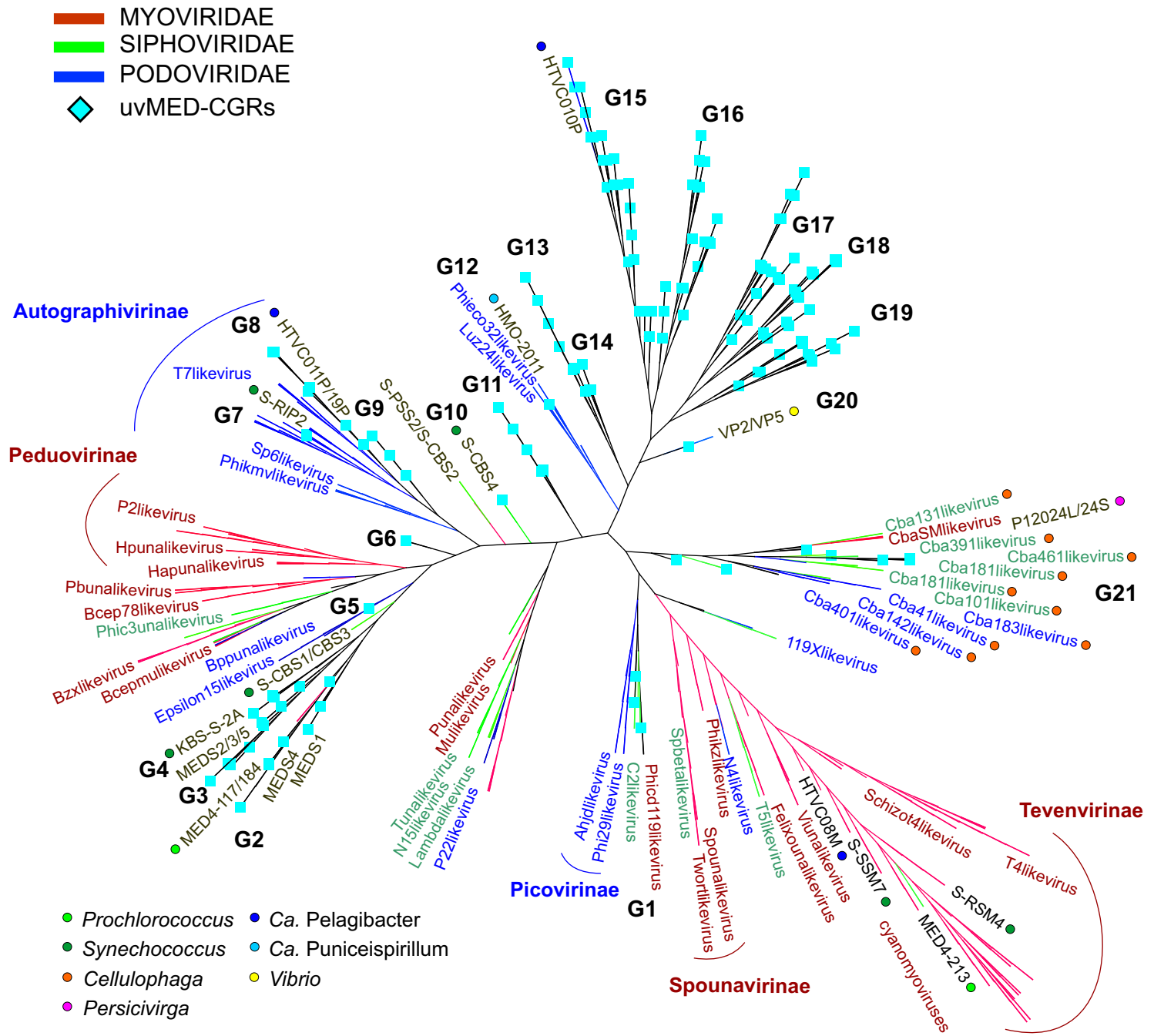

Supplement: Figure S5 — Genomic comparison of novel, complete phage genomes (CGRs), with known tailed phages. This figure is similar to Fig. 2 in the main text but contains genus names of several reference phages. All names were obtained from the ICTV classification (http://www.ictvonline.org). (PDF) [file pgen.1003987.s006.pdf]

*psbA*

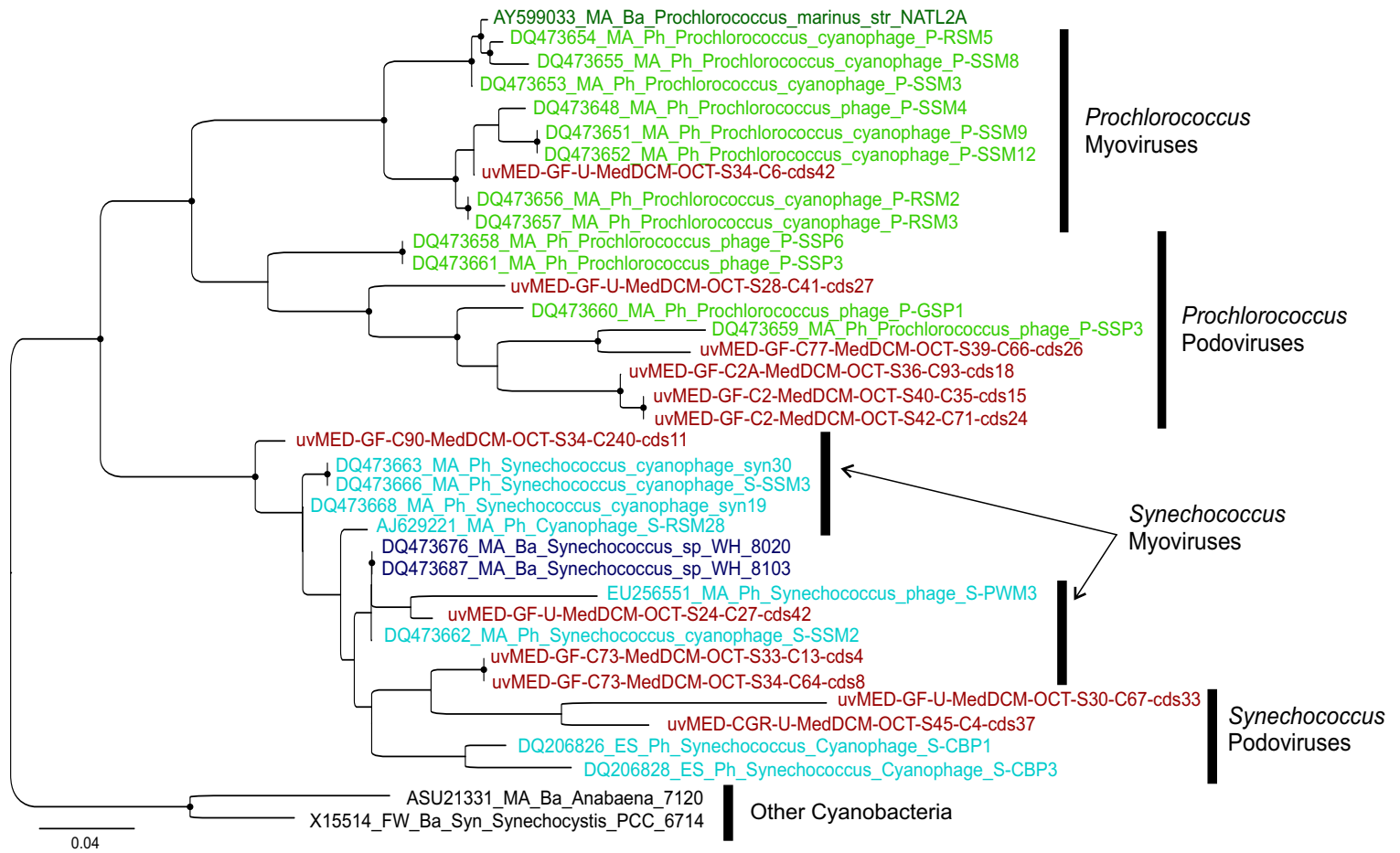

Supplement: Figure S6 — Phylogenetic tree of psbA gene sequences. psbA sequences obtained from cyanobacteria, cultivated cyanophages and fosmid cyanophage contigs are shown in this tree. Sequence names are color coded as follows: Prochlorococcus, dark green; Synechococcus, dark blue; Prochlorococcus cyanophages, light green; Synechococcus cyanophages, light blue; uvMED fosmid contigs, red. Each sequence from GenBank is labeled as follows: accession number_ marine(MA)/estuary(ES)/freshwater(FW)_ Phage(Ph)/bacteria(Ba)_ GenBank description. Bootstrap values of >50% are shown as black circles at each node. Host name and phage morphology is indicated on the right for some clusters. (PDF) [file pgen.1003987.s007.pdf]

*psbD*

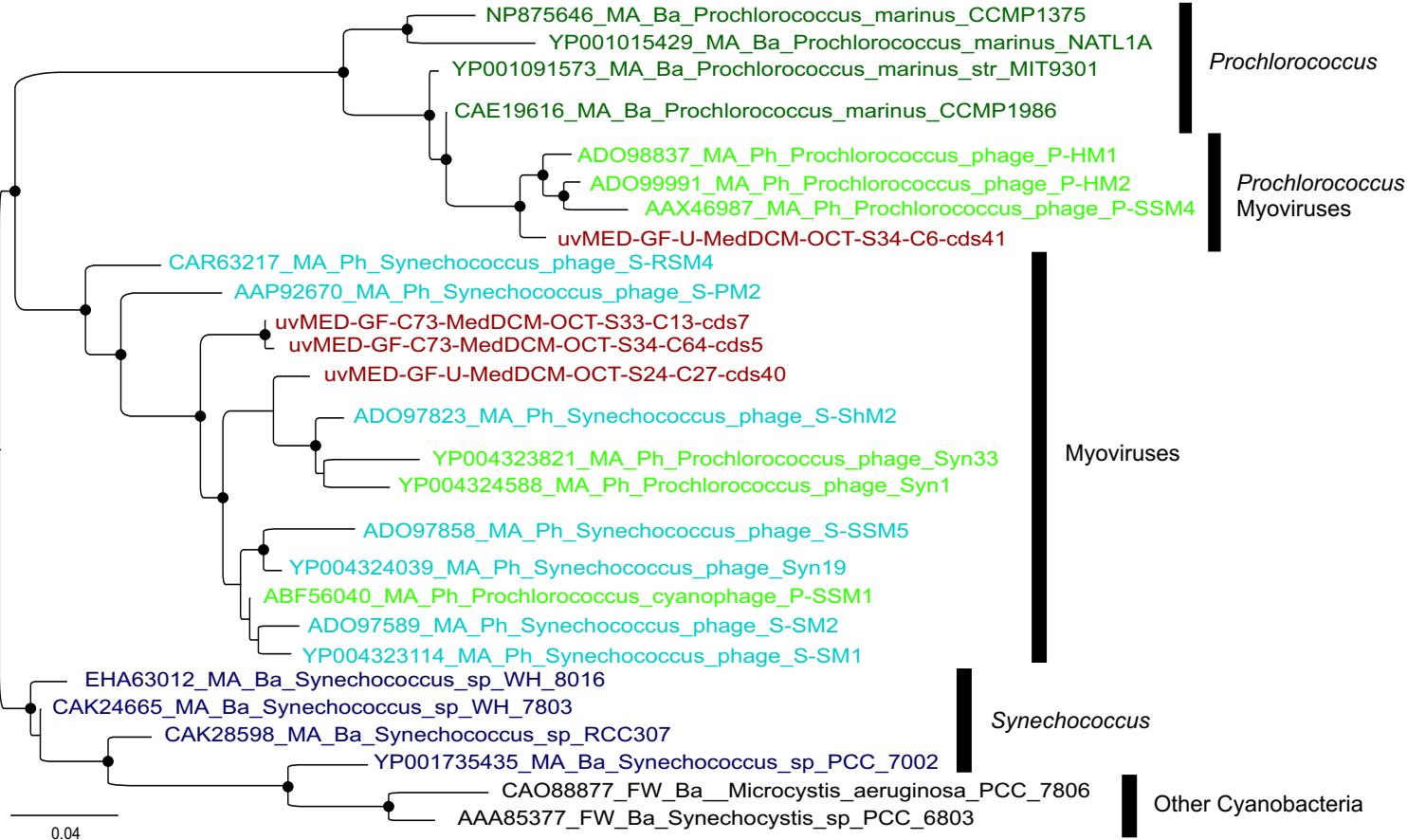

Supplement: Figure S7 — Phylogenetic tree of psbD gene sequences. Several psbD sequences obtained from cyanobacteria, cultivated cyanophages and the uvMED fosmid contigs are shown in this tree. Sequence names are color coded as follows, Prochlorococcus, dark green; Synechococcus, dark blue; Prochlorococcus cyanophages, light green; Synechococcus cyanophages, light blue; uvMED fosmid contigs, red. Each sequence from GenBank is labeled as follows: accession number_ marine(MA)/estuary(ES)/freshwater(FW)_ Phage(Ph)/bacteria(Ba)_ GenBank description. Bootstrap values of >50% are shown as black circles at each node. Clusters of sequences are labeled according to their origin and phage morphology wherever applicable. (PDF) [file pgen.1003987.s008.pdf]

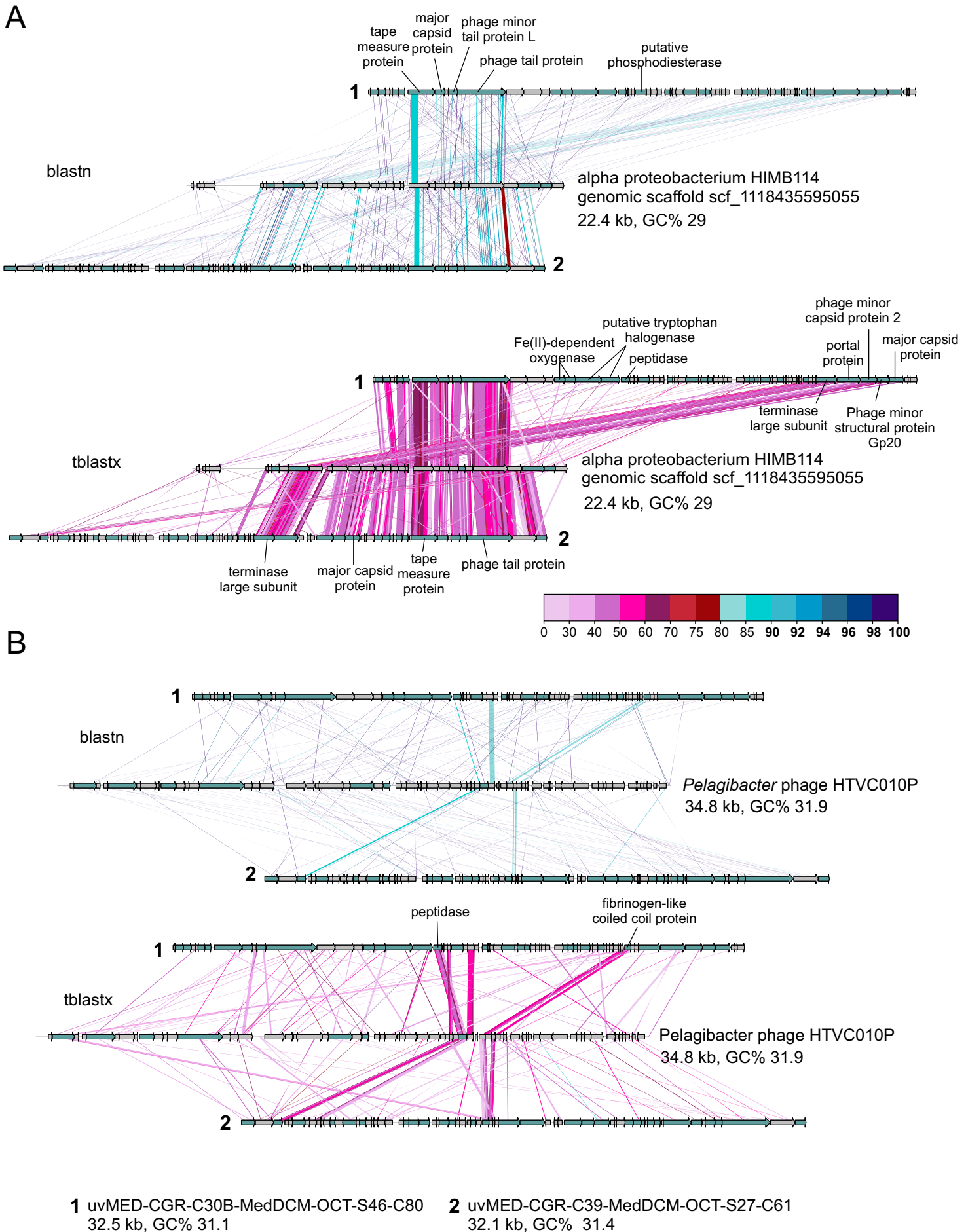

Supplement: Figure S8 — Putative SAR11 CGRs. BLASTN and TBLASTX comparisons of CGRs of group G19 versus. (A) the prophage locus in alpha proteobacterium HIMB114. (B) Ca. Pelagibacter phage HTVC010P. A color key for the %identities is also shown. (PDF) [file pgen.1003987.s009.pdf]

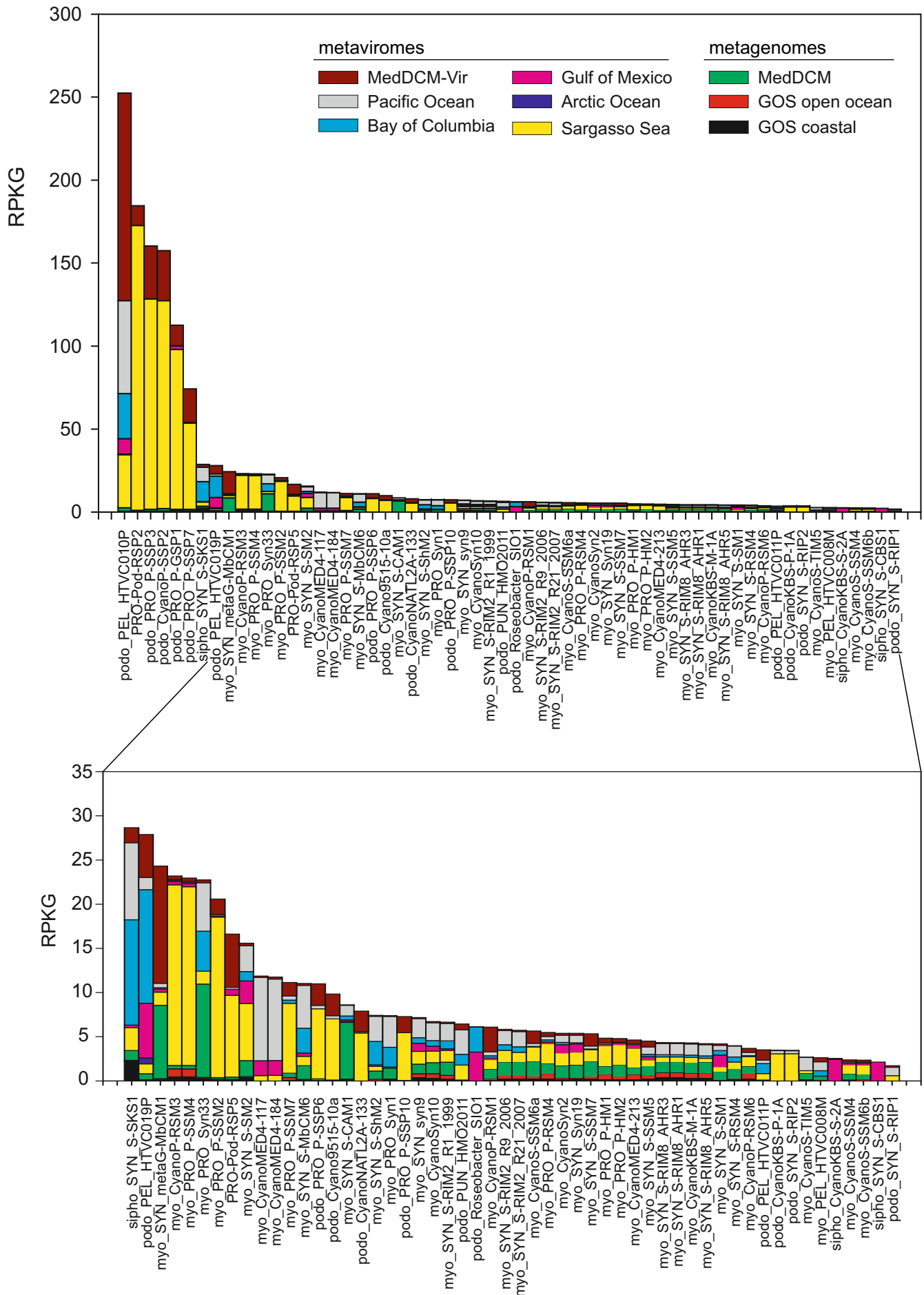

Supplement: Figure S9 — Comparative fragment recruitment of cultivated phage genomes. Number of reads recruited by each, expressed as RPKG (Reads per Kb per Gb) from several metaviromes and metagenomes (color coded). Only hits that had >95% identity, minimum length of 50 bp and e-value<1e-5 were considered in this analysis. A magnified view for the less recruiting phages is also shown below. (PDF) [file pgen.1003987.s010.pdf]

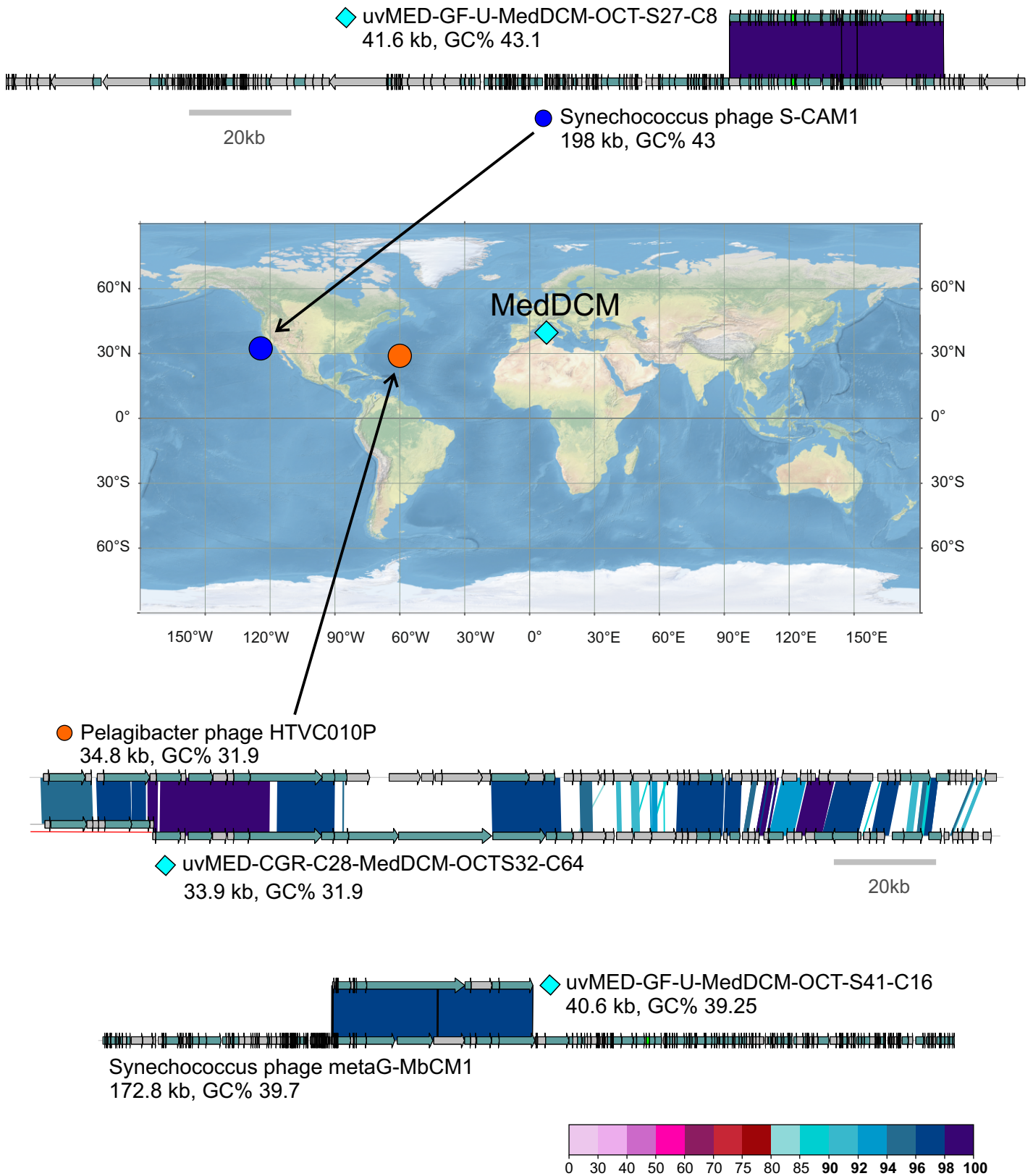

Supplement: Figure S12 — Mediterranean phage contigs identical to cultivated phages isolated from distant geographical locations. Three fosmid contigs are shown, each compared to the genome of a phage that was not isolated from the Mediterranean. All phage genomes and contigs are labeled and their size and GC% is indicated. A color key for the %identity of the alignments is shown in the bottom right corner. Three locations are marked in the world map (blue circle: isolation of Synechococcus phage S-CAM1, orange circle: isolation of Ca. Pelagibacter phage HTVC010P, and blue diamond: Mediterranean. Location information for the Synechococcus phage metaG-MbCM1 was not available. Some gene clusters are shown displaced and underlined in the graphic indicating that they have been moved to improve comparison across all genomes. The map shown in the figure was obtained from www.naturalearthdata.com. (PDF) [file pgen.1003987.s013.pdf]
