# Supplementary material for: Expanding the Marine Virosphere Using Metagenomics
Source: PLoS Genet. 2013 Dec 12;9(12):e1003987. doi: 10.1371/journal.pgen.1003987 (PMC3861242; doi:10.1371/journal.pgen.1003987)
Supplement: Figure S3 — Genomic comparisons of Myoviruses. The three main subfamilies, Tevenvirinae, Spounavirinae and Peduovirinae are indicated in bold. Phages belonging to the same genus are labeled by the Genus name, e.g. Hpunalikevirus, Phikzlikevirus etc. Classification details were obtained from http://www.ictvonline.org. (PDF) [file pgen.1003987.s004.pdf]

Myoviruses

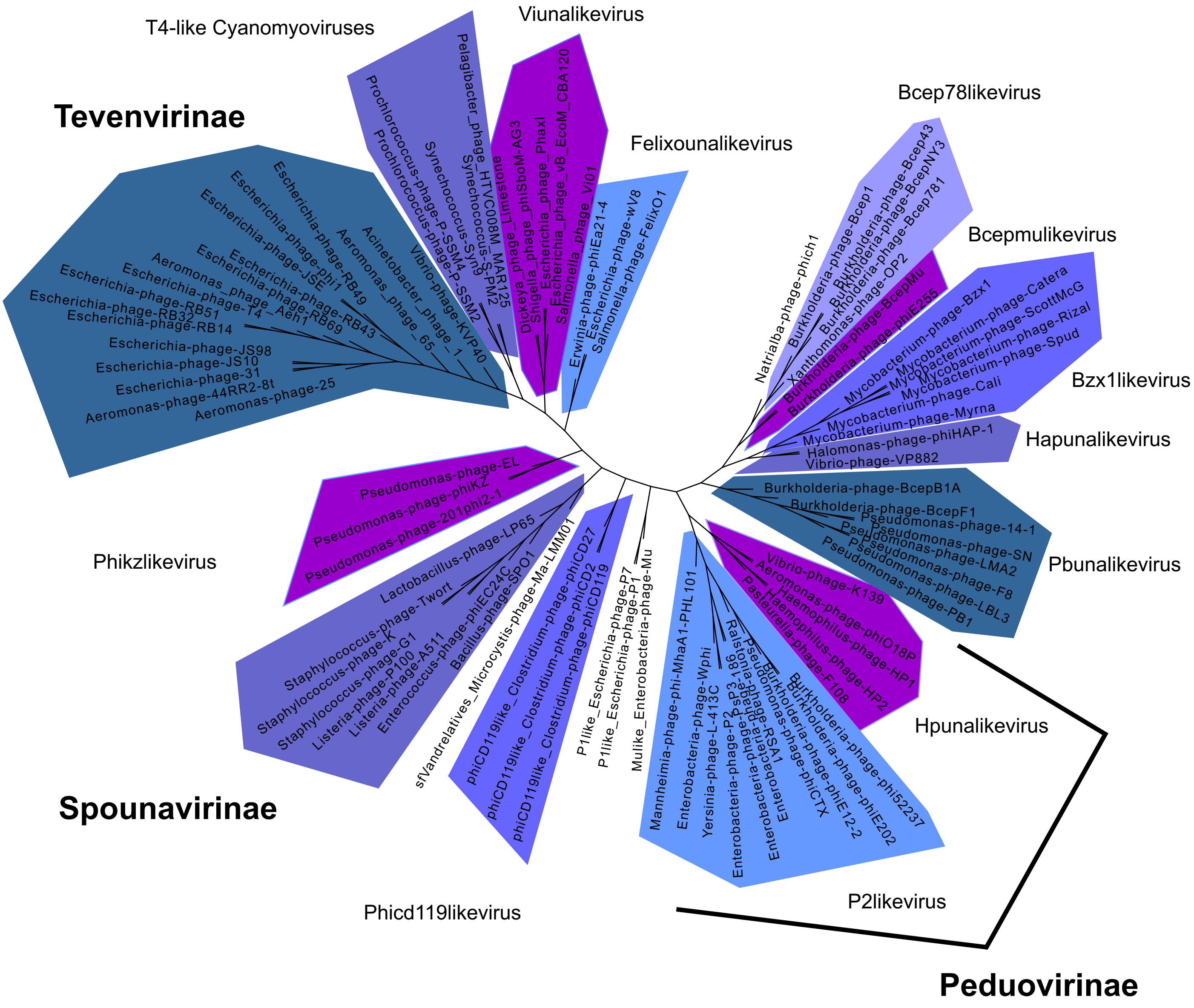
T4-like Cyanomyoviruses
Viunalikevirus
Felixounalikevirus
Bcep78likevirus
Bcepmulikevirus
Bzx1likevirus
Hapunalikevirus
Pbunalikevirus
Hpunalikevirus
P2likevirus
Phicd119likevirus
Phikzlikevirus
Tevenvirinae
Spounavirinae
Peduovirinae
Pelagibacter_phage_HTVC008M
Prochlorococcus-phage-P-SSM4
Prochlorococcus-phage-P-SSM2
Synechococcus-phage-S-PM2
Synechococcus-Syn9
MAR125
Dickeya_phage_Limestone
Shigella_phage_phiSboM-AG3
Escherichia_phage_PhaxI
Escherichia_phage_vB_EcoM_CBA120
Salmonella_phage_Vi01
Erwinia-phage-phiEa21-4
Escherichia-phage-wV8
Salmonella-phage-FelixO1
Escherichia-phage-RB49
Escherichia-phage-phi1
Escherichia-phage-JSE
Escherichia-phage-RB43
Escherichia-phage-RB69
Aeromonas_phage_Aeh1
Escherichia-phage-T4
Escherichia-phage-RB51
Escherichia-phage-RB32
Escherichia-phage-RB14
Escherichia-phage-JS98
Escherichia-phage-JS10
Escherichia-phage-31
Aeromonas-phage-44RR2-8t
Aeromonas-phage-25
Aeromonas_phage_65
Acinetobacter_phage_1
Vibrio-phage-KVP40
Natrialba-phage-phiCh1
Burkholderia-phage-Bcep1
Burkholderia-phage-Bcep43
Burkholderia-phage-BcepNY3
Burkholderia-phage-Bcep781
Xanthomonas-phage-OP2
Burkholderia-phage-BcepMu
Burkholderia-phage-phiE255
Mycobacterium-phage-Bzx1
Mycobacterium-phage-Catera
Mycobacterium-phage-ScottMcG
Mycobacterium-phage-Rizal
Mycobacterium-phage-Spud
Mycobacterium-phage-Cali
Mycobacterium-phage-Myrna
Halomonas-phage-phiHAP-1
Vibrio-phage-VP882
Burkholderia-phage-BcepB1A
Burkholderia-phage-BcepF1
Pseudomonas-phage-14-1
Pseudomonas-phage-SN
Pseudomonas-phage-LMA2
Pseudomonas-phage-F8
Pseudomonas-phage-LBL3
Pseudomonas-phage-PB1
Vibrio-phage-K139
Aeromonas-phage-phiO18P
Haemophilus-phage-HP1
Haemophilus-phage-HP2
Pasteurella-phage-F108
Burkholderia-phage-phi52237
Burkholderia-phage-phiE202
Burkholderia-phage-phiE12-2
Pseudomonas-phage-phiCTX
Enterobacteria-phage-186
Enterobacteria-phage-PsP3
Enterobacteria-phage-P2
Yersinia-phage-L-413C
Enterobacteria-phage-Wphi
Mannheimia-phage-phi-MhaA1-PHL101
Mulike_Enterobacteria-phage-Mu
P1like_Escherichia-phage-P1
P1like_Escherichia-phage-P7
phiCD119like_Clostridium-phage-phiCD119
phiCD119like_Clostridium-phage-phiCD2
phiCD119like_Clostridium-phage-phiCD27
sfVandrelatives_Microcystis-phage-Ma-LMM01
Bacillus-phage-SPO1
Enterococcus-phage-phiEC24C
Listeria-phage-A511
Listeria-phage-P100
Staphylococcus-phage-G1
Staphylococcus-phage-K
Staphylococcus-phage-Twort
Lactobacillus-phage-LP65
Pseudomonas-phage-EL
Pseudomonas-phage-phiKZ
Pseudomonas-phage-201phi2-1
