# Supplementary material for: Expanding the Marine Virosphere Using Metagenomics
Source: PLoS Genet. 2013 Dec 12;9(12):e1003987. doi: 10.1371/journal.pgen.1003987 (PMC3861242; doi:10.1371/journal.pgen.1003987)

C12B cluster

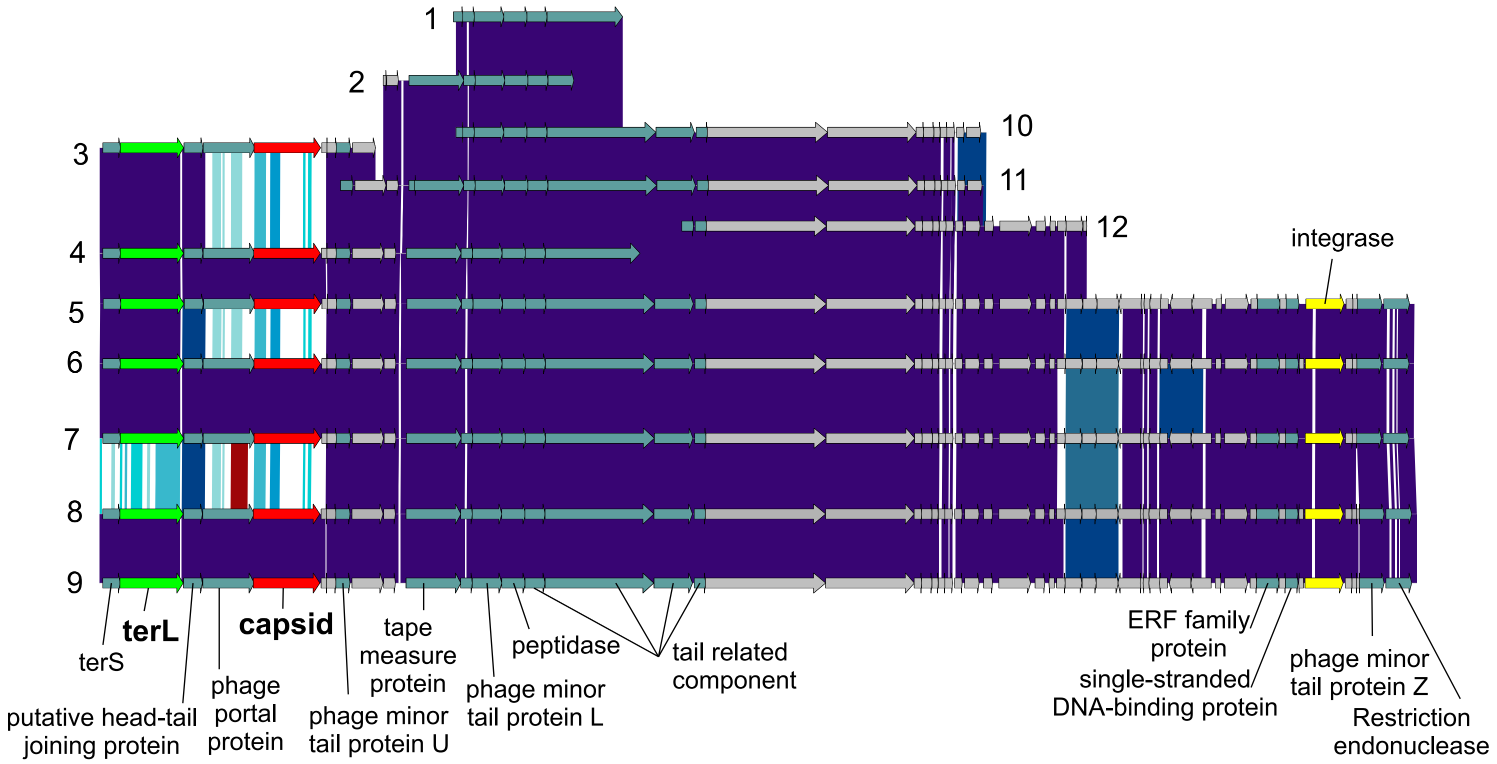

1 uvMED-GF-C12B-MedDCM-OCT-S44-C359 5.1 kb, GC% 31.7
2 uvMED-GF-C12B-MedDCM-OCT-S41-C281 5.7 kb, GC% 31.1
3 uvMED-GF-C12B-MedDCM-OCT-S41-C215 8.3 kb, GC% 33.1
4 uvMED-GF-C12B-MedDCM-OCT-S26-C131 16.3 kb, GC% 32.3
5 uvMED-GF-C12B-MedDCM-OCT-S27-C13 39.7 kb, GC% 31.8
6 uvMED-GF-C12B-MedDCM-OCT-S29-C16 39.6 kb, GC% 31.8
7 uvMED-GF-C12B-MedDCM-OCT-S42-C22 39.6 kb, GC% 31.8
8 uvMED-GF-C12B-MedDCM-OCT-S33-C14 39.7 kb, GC% 31.8
9 uvMED-GF-C12B-MedDCM-OCT-S45-C20 39.7 kb, GC% 31.8
10 uvMED-GF-C12B-MedDCM-OCT-S24-C109 16 kb, GC% 32.1
11 uvMED-GF-C12B-MedDCM-OCT-S39-C115 19.4 kb, GC% 32.1
12 uvMED-GF-C12B-MedDCM-OCT-S41-C174 12.2 kb, GC% 32.6

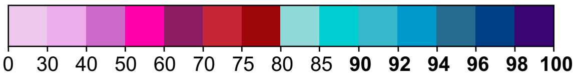

Supplement: Figure S10 — Nearly identical, concurrent phage contigs. A nucleotide comparison of several highly related contigs is shown. Color key for the %identity is shown in the top right corner. All these contigs were clustered together in the cluster C12B. Contigs are labeled by a number (1, 2, 3 etc.) and the full contig names are given below. Selected genes are labeled and colored uniformly. (PDF) [file pgen.1003987.s011.pdf]
