# Supplementary material for: Expanding the Marine Virosphere Using Metagenomics
Source: PLoS Genet. 2013 Dec 12;9(12):e1003987. doi: 10.1371/journal.pgen.1003987 (PMC3861242; doi:10.1371/journal.pgen.1003987)
Supplement: Figure S11 — Concurrent, hybrid phage contigs. A nucleotide comparison of several highly related contigs is shown. Color key for the %identity is shown in the bottom right corner. All these contigs were clustered together in the cluster C10. Contigs are labeled by a number (1, 2, 3 etc.) and the full contig names are given below, along with size and GC%. Selected genes are labeled. (PDF) [file pgen.1003987.s012.pdf]

C10 cluster

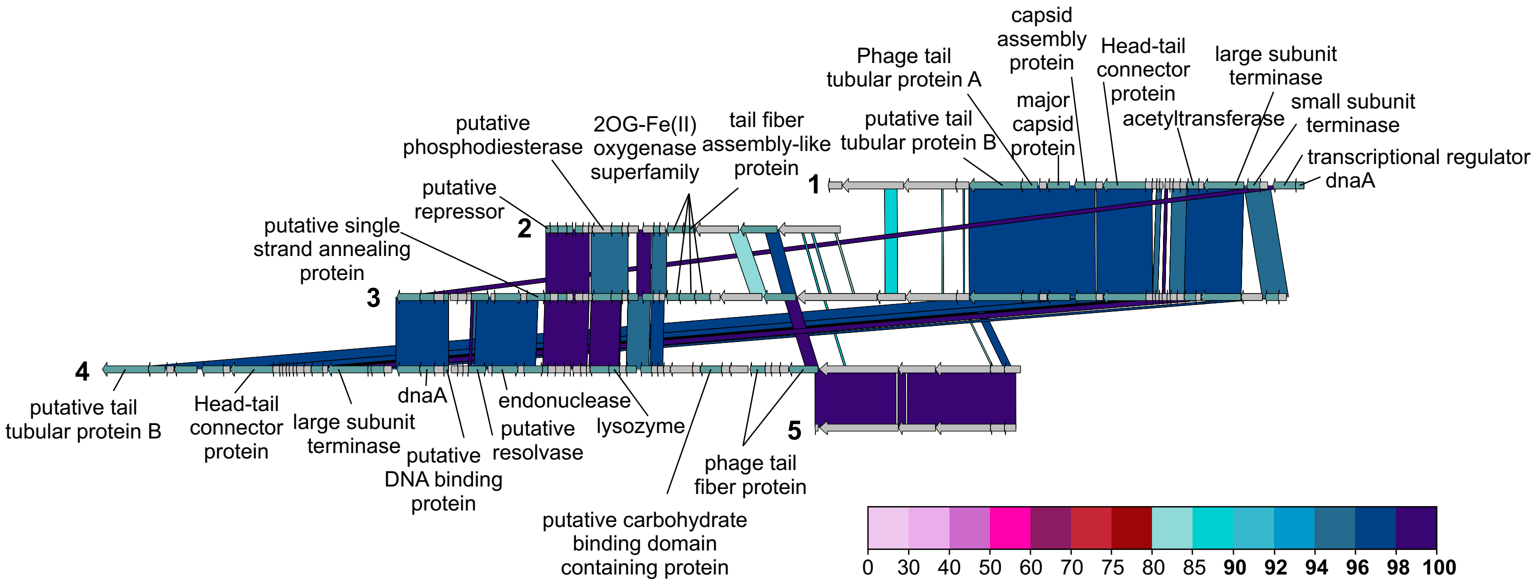

1 uvMED-CGF-C10-MedDCM-OCT-S37-C126; 17.9 kb, GC% 32.8

2 uvMED-CGF-C10-MedDCM-OCT-S37-C174; 11.1 kb, GC% 33.8

3 uvMED-CGR-C10-MedDCM-OCT-S30-C55; 33.7 kb, GC% 32.9

4 uvMED-CGR-C10A-MedDCM-OCT-S46-C61; 34.6 kb, GC% 33.2

5 uvMED-CGF-C10A-MedDCM-OCT-S41-C229; 7.5 kb, GC% 30.9
